# Regulated Cell Death in Urinary Malignancies

**DOI:** 10.3389/fcell.2021.789004

**Published:** 2021-11-12

**Authors:** Zhenyu Nie, Mei Chen, Yuanhui Gao, Denggao Huang, Hui Cao, Yanling Peng, Na Guo, Shufang Zhang

**Affiliations:** Central Laboratory, Affiliated Haikou Hospital of Xiangya Medical College, Central South University, Haikou, China

**Keywords:** urinary malignancies, necroptosis, pyroptosis, ferroptosis, neutrophil extracellular traps

## Abstract

Urinary malignancies refer to a series of malignant tumors that occur in the urinary system and mainly include kidney, bladder, and prostate cancers. Although local or systemic radiotherapy and chemotherapy, immunotherapy, castration therapy and other methods have been applied to treat these diseases, their high recurrence and metastasis rate remain problems for patients. With in-depth research on the pathogenesis of urinary malignant tumors, this work suggests that regulatory cell death (RCD) plays an important role in their occurrence and development. These RCD pathways are stimulated by various internal and external environmental factors and can induce cell death or permit cell survival under the control of various signal molecules, thereby affecting tumor progression or therapeutic efficacy. Among the previously reported RCD methods, necroptosis, pyroptosis, ferroptosis, and neutrophil extracellular traps (NETs) have attracted research attention. These modes transmit death signals through signal molecules, such as cysteine-aspartic proteases (caspase) family and tumor necrosis factor-α (TNF-α) that have a wide and profound influence on tumor proliferation or death and even change the sensitivity of tumor cells to therapy. This review discussed the effects of necroptosis, pyroptosis, ferroptosis, and NETs on kidney, bladder and prostate cancer and summarized the latest research and achievements in these fields. Future directions and possibility of improving the denouement of urinary system tumors treatment by targeting RCD therapy were also explored.

## Introduction

Tumors of the urinary system generally include kidney, bladder and prostate cancer. In 2020, the number of estimated new cases of urinary malignancies in the United States of America (United States) reached 159,120 and 33,820 deaths (Siegel et al., 2020). In particular, approximately 64,000 and 115,000 patients are diagnosed with renal cancer in the United States and Europe, respectively. This malignancy accounts for approximately 5% of all new tumors and causes nearly 15,000 and 49,000 deaths per year in the United States and Europe (Siegel et al., 2017). With an estimated 81,400 new cases and 17,980 deaths in 2020 alone, bladder cancer is the 4th most common and 8th most lethal malignancy among men in the United States (Siegel et al., 2020). With approximately 19,1930 new cases and 33,330 deaths in 2020, prostate cancer is the most prevalent malignancy among men in the United States and has ranked second in the mortality rate of malignant tumors (Siegel et al., 2020). For bladder cancer, the medical expenses caused by bladder cancer in the United States was $US4 billion in 2010, and this expenditure will reach $US5 billion in 2020 (Yeung et al., 2014). China is the biggest developing countries in the world. With economic development, people’s living standards have been continuously improved, and the incidence and mortality of tumors have also increased year by year. In 2015, the number of new cases in every 10,000 person of prostate cancer is 9.5, and this number has risen to 12.5 in every 10,000 person in 2020 (Chen et al., 2016; Wei et al., 2021). The incidence and mortality of prostate cancer have risen from 60.3 thousand and 26.6 thousands in 2015 to 115.4 thousands and 51.1 thousands in 2020, respectively (Chen et al., 2016; Wei et al., 2021). The incidence of kidney cancer and bladder cancer in 2015 are 66.8 thousands and 80.5 thousands, and the mortality of those two type of cancers are 23.4 thousands and 32.9 thousands in 2015, respectively (Chen et al., 2016). Further in-depth research on the occurrence and development of urinary system tumors will benefit patients and save economic resources for the entire society. Basic research on urinary system tumors is still needed, and a substantial breakthrough is expected.

The pathways of cell death are usually divided into accidental cell death (ACD) and regulated cell death (RCD). ACD is an uncontrolled process triggered by accidental injurious stimuli, such as extreme physical temperature, pressure, chemical stress, or osmotic pressure, which exceed the adjustable ability of the cell and thus lead to cell death. The hallmark of ACD is cell ruptured, cellular contents leaked to the extracellular and damaged to the intracellular environment. RCD also shows cell ruptured and cell content leaked (Hitomi et al., 2008), such as necroptosis, pyroptosis, ferroptosis, and neutrophil extracellular traps (NETs). This process involves signal cascades and effector molecules and has unique biochemical, morphological, and immunological consequences. These characteristics can be used by researchers as molecular markers and by clinicians to assess the patient condition. RCD also affects tumor occurrence, progression, death, and treatment sensitivity. Therefore, attention has been focused on the impact of RCD on tumors and its occurrence and extent.

In this work, four types of RCDs in urinary malignancies were discussed to reveal their role in the occurrence and development of urinary system tumors. Current research progress and challenges were also summarized to encourage further research.

## RCDs and the Molecular Mechanism

### Necroptosis

In 2005, Degterev et al. discovered a special form of necrosis regulated by a specific cell signaling pathway and can be inhibited by necrostain-1 (Nec-1) and proposed procedural necrosis, which can be regulated by Nec-1 (Hitomi et al., 2008). Using small interfering RNA (siRNA) screening methods, researchers later proved that receptor interacting serine/threonine protein kinase 1 (RIPK1) is the target of Nec-1 and many genes regulate programmed necrosis via several pathways (Degterev et al., 2008; Grootjans et al., 2017). In 2018, the Nomenclature Committee on Cell Death officially defined necroptosis as programmed necrosis that requires RIPK1, receptor interacting serine/threonine protein kinase 3 (RIPK3), and substrate mixed lineage kinase domain like pseudokinase (MLKL). Independent from cysteine-aspartic proteases (caspase) is another significant feature of necroptosis (Galluzzi et al., 2018).

### The Molecular Mechanism of Necroptosis

Many molecules are involved in necroptosis, and its regulation is precise and complicated. The two current in-depth methods are death receptors-dependent or -independent necroptosis. Tumor necrosis factor-α (TNF-α) is an inflammation-related cytokine with an important role in inflammation. In the absence of pathogen infection, TNF-α can also induce necroptosis in cells (Brault et al., 2018).

#### The Formation of Complex I and II

**FIGURE 1:**
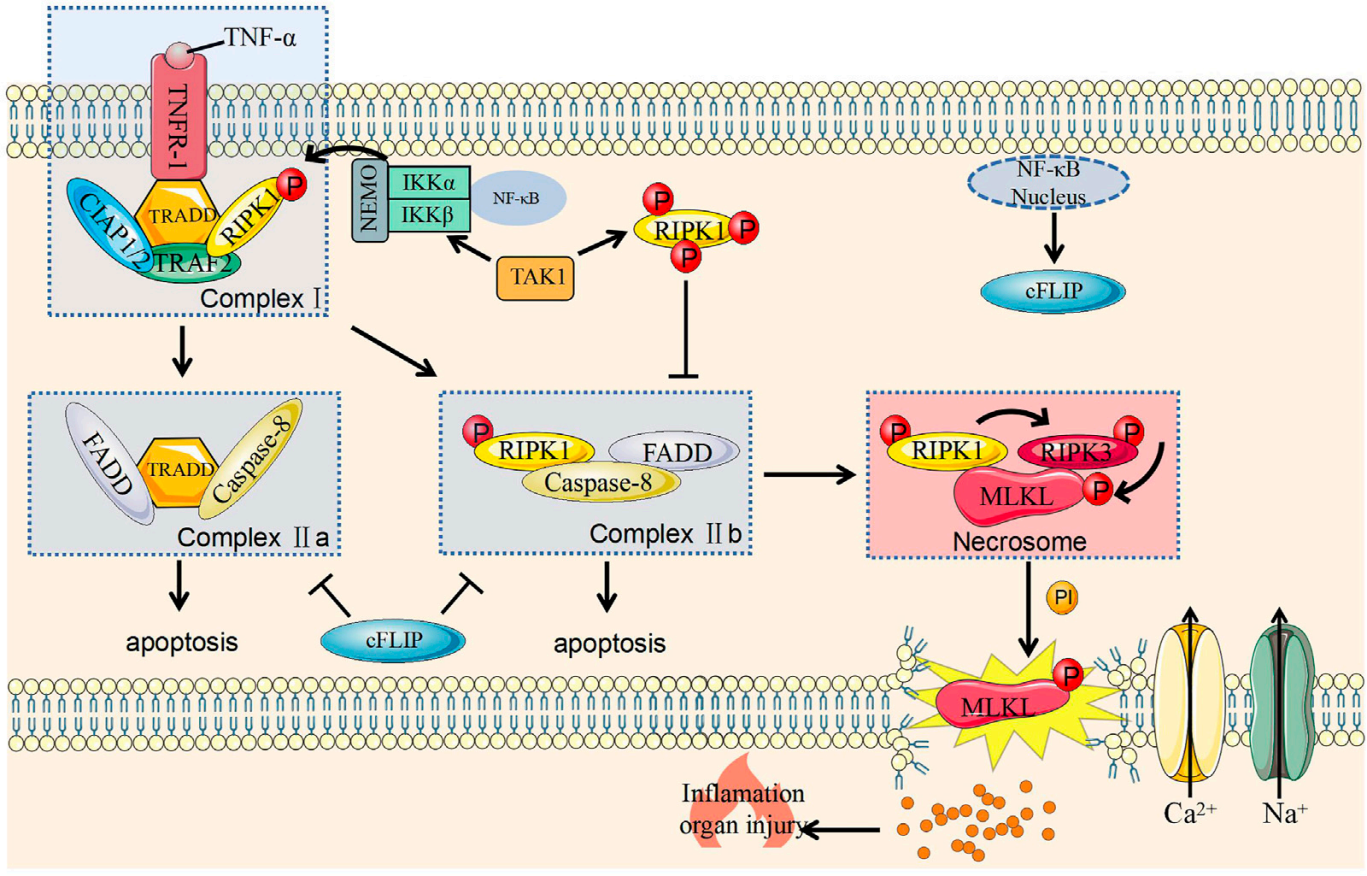
Molecular mechanism of necroptosis and its regulation. Caspase-8, cysteine-aspartic proteases-8; cFLIP, cellular (Fas asscoiated deathe domain like IL-1 converting enzyme)/caspase-8 inhibitior protein; cIAP1/2, cellular inhibitor of apoptosis protein 1 and 2; FADD, Fas-associating protein with a novel death domain; IKKα, IκB kinase α; IKKβ, IκB kinase β; IP, inositol phosphate; MLKL, mixed lineage kinase domain like pseudokinase; NEMO, nucleart factor-κB essential modulator; NF-κB, nuclear factor kappa-B; P, Phosphorylation; TNF-α, tumor necrosis factor-α; TAK1, transforming grow factor-beita actived kinase 1; TNFR1, tumor necrosis factor receptor 1; TRADD, TNF receptor associated death domain; RIPK1, receptor interacting serine/threonine protein kinase 1; TRAF2, TNF receptor associated factor 2; RIPK3, receptor interacting serine/threonine protein kinase 3.

When TNF-α binds to tumor necrosis factor receptor 1 (TNFR1) on the cell membrane, the latter changes its conformation and recruit tumor necrosis factor receptor associated death domain (TRADD) to form a complex with RIPK1. As scaffold proteins, TRADD and RIPK1 continue to recruit tumor necrosis factor-related factor 2 (TRAF2) and cellular inhibitor of apoptosis protein 1 and 2 (cIAP1/2). At this stage, complex I containing TRAD, RIPK1, TRAF2, and cIAP1/cIAP2 has been formed on the cell membrane (Figure 1) (Galluzzi et al., 2017).

Complex I can activate the nuclear factor kappa-B (NF-κB) signaling pathway to promote cell survival and induce inflammation (Hayden and Ghosh, 2014; Dondelinger et al., 2016). This effect is related to the polyubiquitination of RIPK1 protein (Wong et al., 2010). The activation of NF-κB signaling pathway can induce various genes that promote cell survival, including anti-apoptotic genes, such as c-IAP1/2 and intracellular FLICE inhibitory protein (cFLIP) (Dondelinger et al., 2016). cFLIP is a homologous isoform of caspase-8 but lacks caspase enzyme activity. After binding to caspase-8, this protein inhibits the activation of caspase-8 and protect cells from caspase-8 mediated apoptosis (Chan, 2015). And cIAP2 can promote the degradation of NF-κB inhibitory protein IκB, activate NF-κB, and transduces NF-κB molecules into the nucleus to maintain cell survival (Ting and Bertrand, 2016). In summary, complex I is a key checkpoint for cell survival.

The ubiquitination of various components in complex I helps its stabilization on the cell membrane and inhibit the formation of complex II to promote cell survival (Dillon et al., 2012). However, when this process is blocked, the components will fall off the cell membrane and enter the cytoplasm to form complex II with two forms. The formation of complex IIa is related to the damage of NF-κB-dependent cell death checkpoints (Ting and Bertrand, 2016) and that complex IIb is related to the inhibition of RIPK1 polyubiquitination in complex I. RIPK1 also falls off the cell membrane and recruits and activates FADD and caspase-8 to cause cell apoptosis; this process is called RIPK1-dependent cell apoptosis (Dondelinger et al., 2014) (Figure 1).

#### The Formation of Necrosome and the Execution of Necroptosis

RIPK3 is an important protein that causes necroptosis and can be hydrolyzed by caspase-8 (Chan, 2015). Its deletion can also save embryo from death caused by FADD deletion (Chan, 2015). Therefore, RIPK3 has an important role in necroptosis. Necrosome is composed of combined RIPK1 and RIPK3 (Dondelinger et al., 2014) and could phosphorylate and activate MLKL (Kreuz et al., 2001). The specific mechanism of MLKL in necroptosis has not been fully revealed. MLKL phosphorylation is believed to transfer onto the cell membrane and then directly or indirectly destroy this structure (Kreuz et al., 2001; Blackwell et al., 2013; Cai et al., 2014; Chen et al., 2014; Quarato et al., 2016). This protein also activates sodium and calcium channels on the membrane, leading to cation influx and ultimately to necroptosis (Kreuz et al., 2001; Kaiser, 2011; Cai et al., 2014; Chen et al., 2014; Murphy et al., 2018) (Figure 1).

### Pyroptosis

Pyroptosis was proposed in 2001 and used to describe a kind of cell death mediated by caspase-1 (Cookson and Brennan, 2001). However, researchers have preliminarily reported this process (Wang Y. et al., 2017), and some have discovered that the “apoptosis” caused *Shigella Castellani* is mediated by activating caspase-1 in the host cell (Rathinam and Fitzgerald, 2016). Knocking out caspase-1 can prevent this cell death (Zychlinsky et al., 1992). Pyroptosis is not always accompanied with caspase-1 activation (Galluzzi et al., 2018) and has different morphology and molecular mechanism compared with other pathways. Its characteristic performance is being a type of cell death that triggers inflammatory response. In addition to cytoskeletal protein degradation, membrane perforation or membrane lysis, and efflux of intracellular pro-inflammatory substances and inflammatory responses, pyroptosis in cells is manifested by nuclear agglutination, DNA fragmentation markers and positive annexin V positive (Hilbi et al., 1997). Although pyroptosis and necrosis both exhibit membrane rupture and inflammation, the cell membrane of pyropototic cell is gradually dissolved, the cellular contents are released slowly, and the whole process is regulated by the program; by contrast, the plasma membrane of necrosis cells is blast-like, and the process is usually caused by violent and severe external stimulus (Galluzzi et al., 2018). Apoptotic cells also have chromatin condensation, but the DNA is fragmented, the cell membrane remains structurally intact without leakage of cellular contents, and the focal cells have nuclear integrity but lacked of the DNA ladder; these phenomena are accompanied by cell membrane rupture, leakage of cellular contents, and inflammatory responses (Hersh et al., 1999) (Table 1).

**TABLE 1:** Difference among of the five forms of cell death.

	Characteristic	Apoptosis	Necrosis	Pyroptosis	Necroptosis	Ferroptosis
Morphology	Cell lysis	**×**	**√**	**√**	**√**	**√**
	Cell swelling	**×**	**√**	**√**	**√**	**×**
	Membrance perforation	**×**	**√**	**√**	**√**	**×**
	Membrane blebbing	**√**	**×**	**√**	**×**	**×**
	DNA fragmentation	**√**	**√**	**√**	**√**	**×**
	Intact nuclear	**×**	**×**	**√**	**×**	**√**
	Mitochondrial atrophy	**×**	**×**	**×**	**×**	**√**
Mechanism	Caspase-1 activation	**×**	**×**	**√**	**×**	**×**
	Caspase-3 activation	**√**	**×**	**√**	**×**	**×**
	GSDMD activation	**×**	**×**	**√**	**×**	**×**
	RIP3 activation	**×**	**×**	**×**	**√**	**×**
	MLKL activation	**×**	**×**	**×**	**√**	**×**
Denouement	Inflammation	**×**	**√**	**√**	**√**	**√**
	RCD	**√**	**×**	**√**	**√**	**√**

Caspase-1, cysteine-aspartic proteases-1; Caspase-3, cysteine-aspartic proteases-3; MLKL, mixed lineage kinase domain like pseudokinase; GSDMD, gasdemin D; RCD, regulated cell death; RIPK3, receptor interacting serine/threonine protein kinase.

#### Molecular Mechanisms of Pyroptosis

Pyroptosis was first thought to be caspase-1-dependent. With research progress, this process was confirmed to not require caspase-1 and is currently believed to have three main pathways.

##### Caspase-1 Dependent Pyroptosis

**FIGURE 2:**
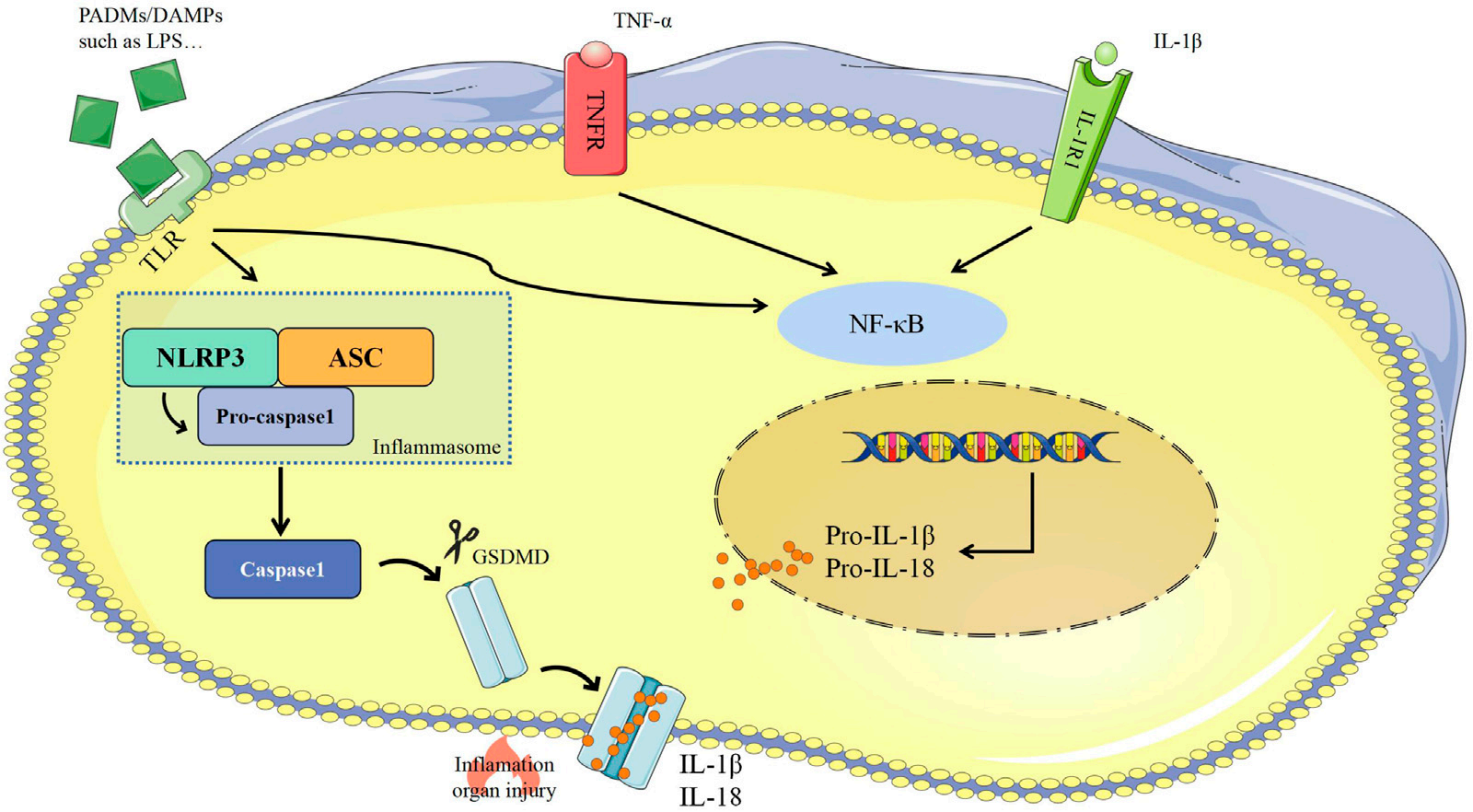
Caspase 1-dependent pyroptosis. ASC, apoptosis-associated speck-like protein; Caspase-1, cysteine-aspartic proteases-1; DAMPs, damage-associated molecular patterns; GSDMD, gasdemin D; IL-1β, interleukin-1β; IL-18, interleukin-18; IL-1R, interleukin-1 receptor; NLRP3, NOD-, LRR-, and pyrin domain-containing protein-3; LPS, lipopolysaccharide; NF-κB, nuclear factor kappa-B; PAMPs, pathogen-associated molecular patterns; TLR, Toll like receptor; TNF-α, tumor necrosis factor-α; TNFR1, tumor necrosis factor receptor.

Pathogen- or damage-associated molecular patterns (PAMPs/DAMPs) are usually secreted from pathogenic microorganisms, directly stimulate cells, and activate NF-κB via pattern recognition receptors (PRRs) (Takeuchi and Akira, 2010). Activated of NF-κB combines with multiple proteins to assemble inflammasomes which includes PRRs such as nucleotide-binding oligomeric domains (NOD)-, leucine-rich repeat domains, (LRR)-, and pyrin domain (PYD)-containing protein 3 (NLRP3), a junctional protein such as apoptosis-associated speck-like protein (ASC), and pro-caspase-1 (Takeuchi and Akira, 2010). Inflammasomes could activate caspase-1 to shear gasdemin D (GSDMD) into the N-terminal GSDMD (GSDMD-NT) that induce cell lysis, release IL-1β and IL-18, and elevate the extent and scale of the inflammatory response (Wilson et al., 1994; Dinarello, 2009; Aglietti et al., 2016; Ding et al., 2016; Liu and Lieberman, 2017). GSDMD is a common substrate for all inflammatory caspases, is cleaved by caspase-1, caspase-4, caspase-5 and caspase-11 and then acquired perforating activity (Liu and Lieberman, 2017) (Figure 2).

##### Caspase-11 Dependent Pyroptosis

**FIGURE 3:**
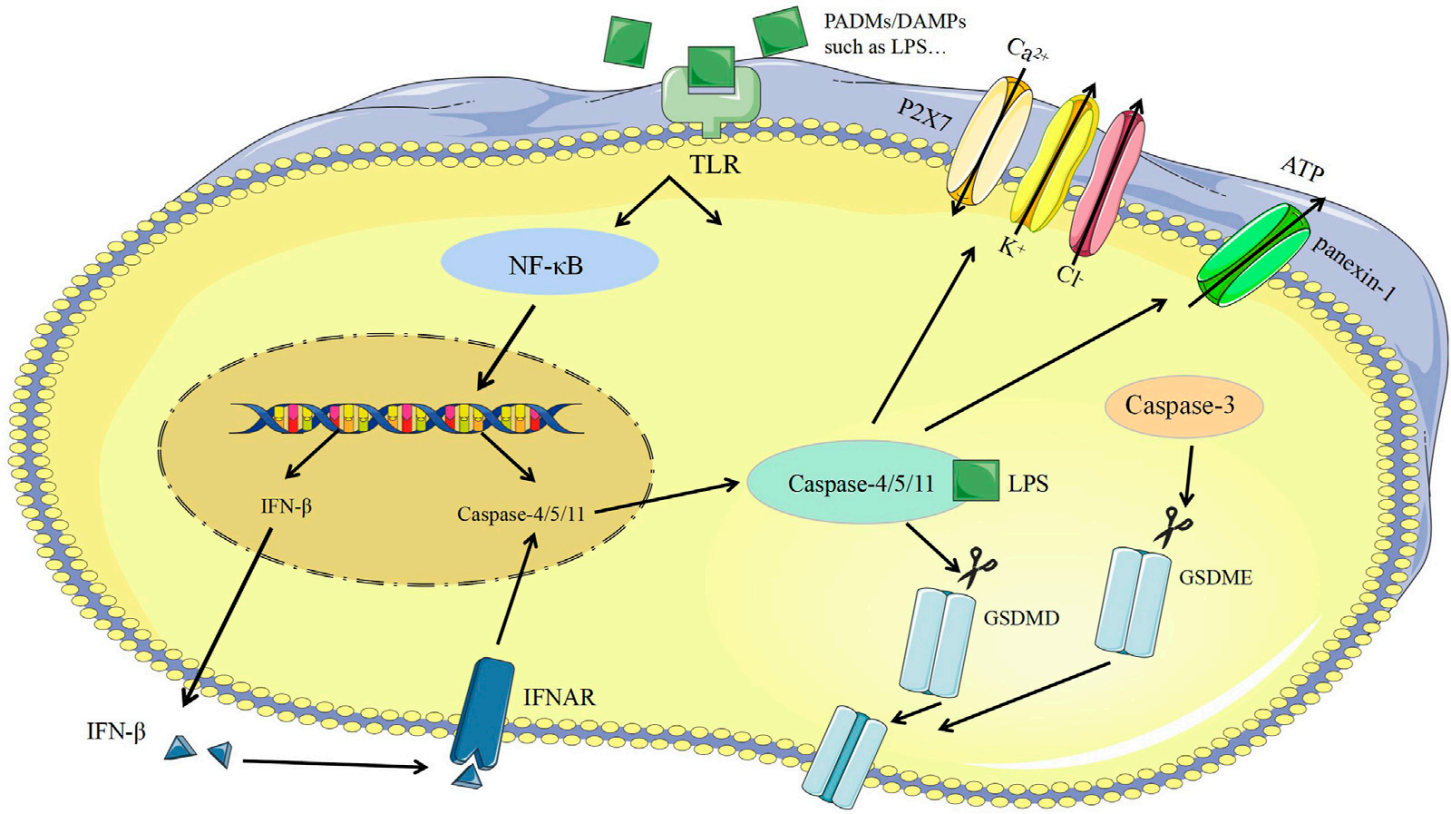
Caspase-1-independent pyroptosis. ATP, Adenosine triphosphate; Caspase-3, cysteine-aspartic proteases-3; Caspase-4, cysteine-aspartic proteases-4; Caspase-8, cysteine-aspartic proteases-8; Caspase-11, cysteine-aspartic proteases-11; DAMPs, damage-associated molecular patterns; GSDMD, gasdemin D; GSDME, gasdemin E; IFN-β, Interferon-β; IFNAR, interferon-α/β receptor; LPS, lipopolysaccharide; NF-κB, nuclear factor kappa-B; PAMPs, pathogen-associated molecular patterns; P2X7, purinergic receptor; TLR, Toll like receptor.

The released Lipopolysaccharide (LPS) of the bacteria could activate and bind with caspase-11 to induce pyroptosis (Viganò and Mortellaro, 2013). On the one hand, caspase-11 can directly cleave GSDMD to induce pyroptosis and promote the assembly of NLRP3 inflammasomes, the activation of pro-caspase-1, and the maturation of IL-1β. On the other hand, caspase-11 can be specifically bind with LPS and then promote the activation of panexin-1, leads to the efflux of intracellular ATP, promotes the activation of P2X purinoceptor 7 (P2X7) to the open of ion channels for the efflux of K^+^ and Cl^−^, meanwhile, Ca^2+^ inflow causes membrane to rupture and inflammatory response (Takeuchi and Akira, 2010; Swanson et al., 2019) (Figure 3).

##### Caspase-3 Dependent Pyroptosis

Certain apoptotic promoted drugs can transform caspase-3 dependent cell apoptosis into caspase-1 dependent pyroptosis (Orning et al., 2018; Sarhan et al., 2018; Wang et al., 2018; Yang et al., 2018; Tang et al., 2021). For example, when the gene of GSDMD of HeLa cells is knocked out and GSDMD with the caspase-3 restriction site is replaced at the same time, HeLa cells can change from apoptotic to pyroptotic (Liu and Lieberman, 2017). When the cells were treated with chemotherapeutics, the high expression of gasdemin E (GSDME) could also convert apoptosis into pyroptosis (Zychlinsky et al., 1992). GSDME is a conserved protein in the GSDM family, and caspase-3 restriction sites exist in its N-terminal and C-terminal domains (Hilbi et al., 1997; Wang et al., 2018; Tang et al., 2021). Once GSDME is cleaved, its N-terminus can specifically bind to phosphatidylinositol-4,5-bisphosphate on the cell membrane, thus causing plasma membrane perforation and inflammatory substances release, and eventually pyroptosis (Hersh et al., 1999; Wang et al., 2018) (Figure 3).

### Ferroptosis

In 2003, a new compound, erastin, was reported to cause the death of cancer cells. However, this cell death is quite different from all the kind of cell death currently known at that time. In this process no apoptosis body is formed, DNA is destroyed, and caspase family molecules are activation and could not be blocked by caspase inhibitors (Dixon et al., 2012). In 2012, a concept of iron-dependent RCD was proposed on the basis of previous research and officially named as ferroptosis (Li et al., 2020). The main morphological changes of ferroptosis include mitochondrial atrophy, such as increased mitochondrial membrane density, remarkably reduction in mitochondrial ridges, rupture of mitochondrial membranes, intact nuclei that are normal in size without chromatin condensation, and cell membrane with increased density but no blebbing (Table 1) (Dolma et al., 2003; Yagoda et al., 2007; Yang and Stockwell, 2008). In addition to these morphological characteristics, ferroptosis also has unique biochemical features often used as evaluated indicators to judge this process in scientific research. The mainly distinctive features include increased the concentration of intracellular Fe^2+^ and reactive oxygen species (ROS), decreased cysteine intake, depletion of glutathione, and increased levels of hydroxyl free radicals and lipid hydroperoxides (LOOH) (Dolma et al., 2003; Yagoda et al., 2007). Owing to its unique metabolic characteristics for Fe^2+^ and ROS, the use of iron chelating agents (such as deferoxamine) or antioxidants could inhibit this ferroptosis (Yang and Stockwell, 2008).

#### The Molecular Mechanism of Ferroptosis

**FIGURE 4:**
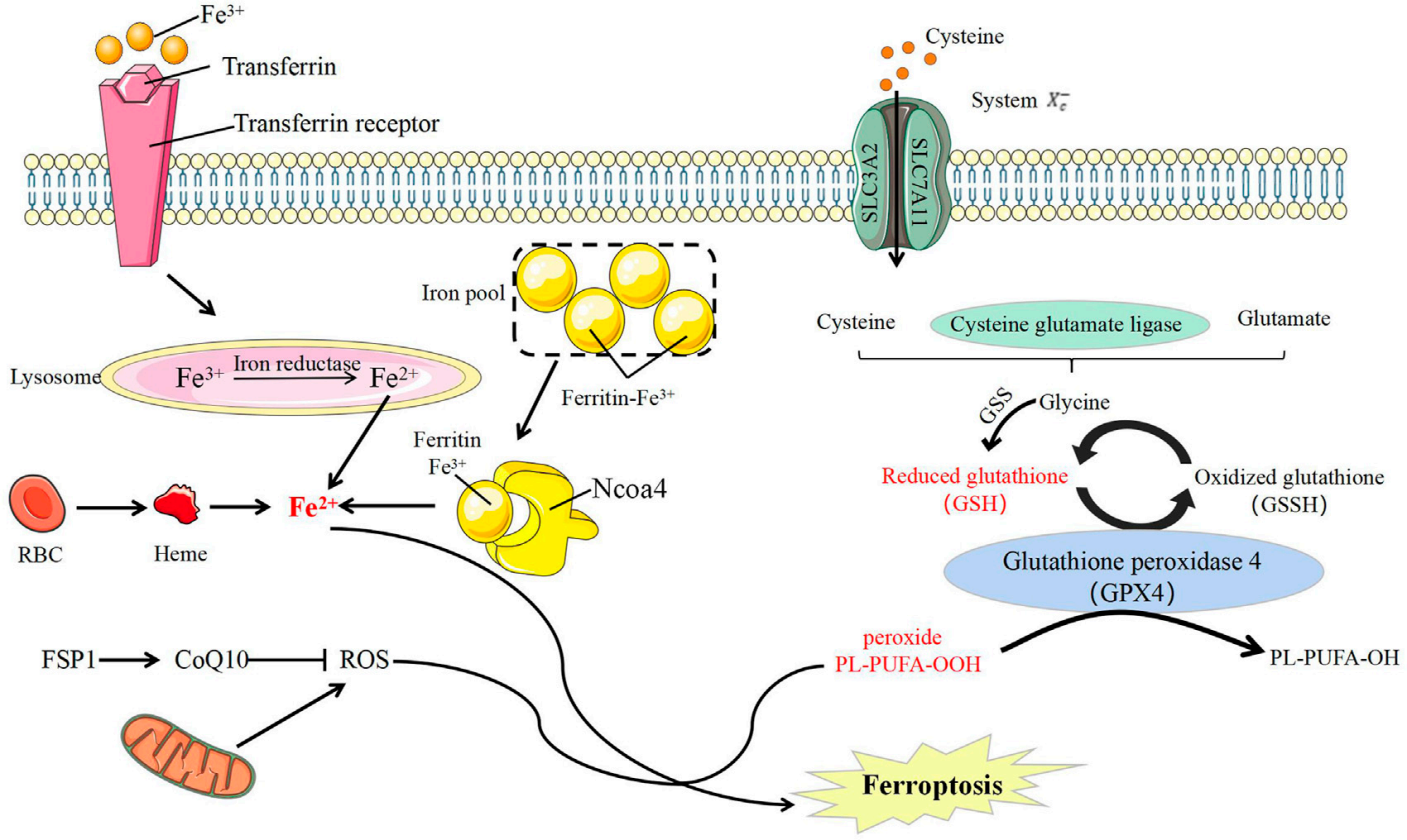
Ferroptosis molecule and its regulation. CoQ10, Coenzyme Q10; FSP1, Ferroptosis-suppressor-protein 1; GSS, glutathione synthetase; NCOA4, nuclear receptor coactivator-4; RBC, red blood cells; ROS, reactive oxygen species; PL, Phospholipid; PUFA, polyunsatureated fatty acid; SLC3A2, solute carrier 3A2; SLC7A11, solute carrier 7A11; System Xc-, the cystine/glutamate antiporter system.

Ferroptosis is mainly regulated by the metabolism of iron, lipid and amino acid. The most influential factor is glutathione peroxidase 4 (GPX4), a membrane lipid repair enzyme that can convert glutathione into oxidized glutathione to limit cytotoxic lipid peroxidation (Seiler et al., 2008; Tang and Kroemer, 2020). The use of GPX4 inhibitors or knockdown of GPX4 can induce ferroptosis in cells, and this process can also be rescued by iron chelating agents (Yant et al., 2003; Yang et al., 2014; Ingold et al., 2018). (Figure 4).

Fe^2+^/Fe^3+^ participates in ROS formation and causes ferroptosis. Intracellular ferritin can release Fe^2+^ through autophagy, and nuclear receptor coactivator-4 (NCOA4) acts as an adaptor protein to mediate this process (Yang et al., 2014). Hence, NCOA4 regulates the sensitivity of cells to ferroptosis by regulating intracellular iron ions. NCOA4 Overexpression can increase the degradation of ferritin and promote the occurrence of ferroptosis (Yang et al., 2014). Iron is a direct factor affecting ROS production. Heme oxygenase-1 (HO-1) can catalyze the degradation of heme to produce free iron (Rouault, 2005), and its overexpression can accelerate ferroptosis induced by erastin (Hentze et al., 2010).

Lipid peroxide is a member of ROS and the ultimate executor of ferroptosis (D’Herde and Krysko, 2016). ROS, including peroxides, superoxides, singlet oxygen and free radicals, is a class of molecules with partially reduced oxygen and cause cell death by damaging DNA, RNA, and lipid molecules (Doll et al., 2019) In ferroptosis, the accumulation of lipid peroxides, especially phospholipid peroxides, is considered to be a landmark event (Gaschler and Stockwell, 2017). Lipid peroxides can cause damage in many ways. On the one hand, lipid peroxides could decompose into ROS and amplify lipid peroxidation. On the other hand, lipid peroxides could change the physical structure of the membrane and disrupt the metabolism of intracellular substances (Yang et al., 2016; Kagan et al., 2017). The main damage of lipid peroxidation is by polyunsatureated fatty acid (PUFA) and phosphatidylethanolamines (PE) oxidative decomposition (Yang et al., 2016; Kagan et al., 2017). PUFA and PE are important components of biomembrane (Lin et al., 2018), and deeply affects the proliferation, differentiation, immunity and other biological functions of cells (Bersuker et al., 2019) (Lin et al., 2018).

As a substrate of GPX4, glutathione (GSH) is a key factor in anti-oxidative stress (Woo et al., 2015). Cystine/glutamate antiporter (system Xc^−^, xCT), a specific transporter required for glutamate and cysteine to enter and exit cells, is composed of glycosylated solute carrier 3A2 (SLC3A2) and non-glycosylated SLC7A11 through disulfide linkages (Li et al., 2020; Reichert et al., 2020). Inhibiting the amino acid imbalance caused by xCT can cause ferroptosis (Gao et al., 2015). The high level of glutamate concentration out of the cells can also inhibit xCT and then induce ferroptosis (Maiorino et al., 2018; Li et al., 2020). GSH can be used as an electron donor to convert toxic phospholipid peroxide into non-toxic phosphatidyl alcohol oxidized glutathione under the action of GPX (Bridges et al., 2011). Drugs such as RSL3 (Yagoda et al., 2007) and hexamethylmelamine (Liu et al., 2021), or interference with GPX4 expression (Lin et al., 2018) can induce and promote ferroptosis. Moreover, this process can be induced by consumption GSH that will prevent GPX4 to lose its effect (Yant et al., 2003; Li et al., 2020).

### NETs

**FIGURE 5:**
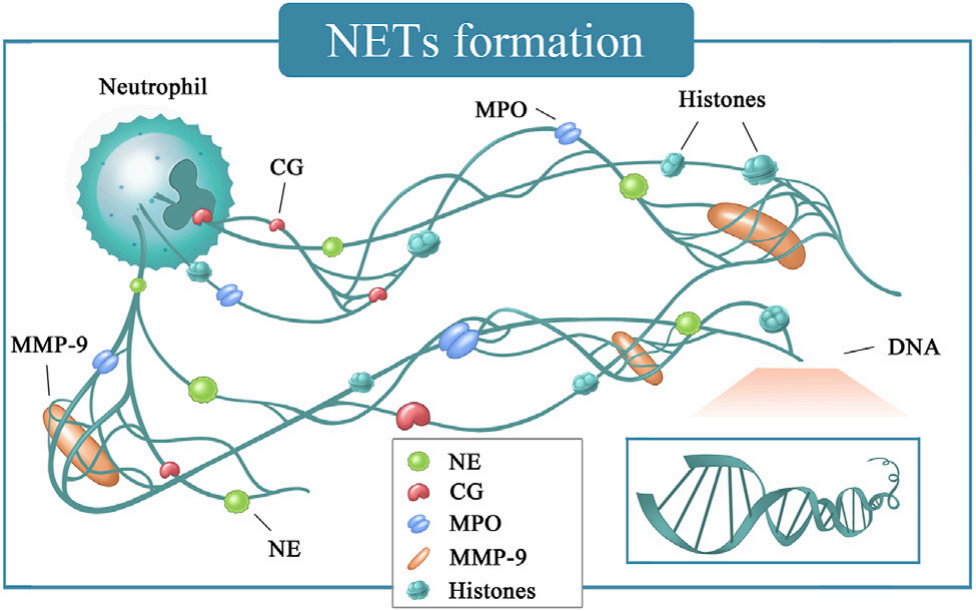
NETs formation. CG, cathepsin G; DNA, deoxyriboNucleic acid; MMP-9, matrix metalloproteinase-9; MPO, myeloperoxidase; NE, neutrophil elastase.

Neutrophils are important members of non-specific immunity and are involved in resisting and killing pathogenic microorganisms, removing necrotic tissues, and repairing the defects (Colotta et al., 2009). These cells and their components are involved in almost every process of tumor progression and metastasis (Brinkmann et al., 2004). For example, neutrophils can secrete various substances affecting the tumor microenvironment and promote tumor metastasis and invasion (Fuchs et al., 2007; Demers et al., 2016; Castanheira and Kubes, 2019). NETs are a fibrous network structure released by neutrophils under the action of stimulating factors and composed of de-aggregated chromatin a various protein particles (Mizuno et al., 2019). NETs were originally thought to be a potential sterilization mechanism that can capture and organize the spread of multiple pathogens in host defense against infection (Mizuno et al., 2019). In addition to the loose backbone made of DNA, the components include matrix metalloproteinase-9 (MMP-9), neutrophil elastase (NE), cathepsin G (CG), and myeloid peroxidase (MPO) surround and attach (Figure 5) (Mizuno et al., 2019). NETs are formed due to the release of intracellular substances after the death of neutrophils. This type of death differs from apoptosis or necrosis and is named NETosis (Wu et al., 2019). A few minutes after neutrophils are activated by external stimuli, they began to flatten, the nucleus lobules decrease until they disappeared, chromatin agglomerated, the inner and outer layers of the nuclear membrane separate from each other, and the granules in the cytoplasm disintegrate. The nuclear membrane then divides into small vesicles, and the nucleus and cytoplasm fuse. At this time, the cells gradually become round and shrink in size (Table 1). Finally, the cell membrane ruptures and releases NETs (Amulic et al., 2017). At present, NETosis is divided into two ways according to whether it needs to rely on nicotinamide adenine dinucleotide phosphate (NADPH).

#### The Anti-Tumor Effects of NETs

Necroptosis, pyroptosis, and ferroptosis all occur on the tumor cells and thus may eventually promote or inhibit tumor growth. NETosis occurs on neutrophils in the tumor microenvironment (TME) and regulates tumor growth by releasing NETs, which are common in many types of tumors. For example, NETs are highly common in lung cancer or osteosarcoma (Reggiani et al., 2017), and the neutrophils have stronger ability to form and release NETs than normal tissue (Acuff et al., 2006; Reggiani et al., 2017; Huang, 2018). MMP-9, which is expressed by various cells, can promote cell migration and angiogenesis (Lerman and Hammes, 2018) and is also involved in different tumor pathophysiological processes (Najmeh et al., 2017). For example, in pancreatic cancer or Lewis lung cancer, MMP-9 can promote tumor growth and development, invasion, and metastasis (Amulic and Hayes, 2011). The NE in NETs can directly promote tumor cells proliferation and distant metastasis (Erpenbeck and Schön, 2017). NETs can also form a physical barrier between tumor cells and immune cells through their special network structure to inhibit the anti-tumor functions of other immune cells (Boone et al., 2015).

Current research on NETs conducing tumor metastasis is quite extensive. This function is mainly accomplished in five aspects: 1) NETs degrade the extracellular matrix. For example, MMP-9 and CG can degrade the extracellular matrix to prepare for tumor metastasis (Wilson et al., 2010; Park et al., 2016; Cabel et al., 2017); 2) NETs capture circulating tumors cells (CTCs), cells that are shed from the primary tumor site or metastasis, entering the vasculature, and thus escape from immune attack. CTCs has important clinical significance in tumor metastasis and huge potential as prognostic markers of metastatic tumors (Li et al., 2010). The 3D structure of NETs can capture CTCs, facilitate its adhesion, and promote metastasis (Li et al., 2010). At the same time, NETs can directly promote the adhesion of CTCs to the vascular wall, and the breaks through the vascular walls to reach distant organs to form new metastases (Acuff et al., 2006; Guo et al., 2012; Kolaczkowska et al., 2015). 3) NETs promote immune evasion. On the one hand, they can form obstruction between immune cells and CTCs (Pieterse et al., 2017); on the other hand, NETs and their dissolved products can also inhibit the normal functions of immune cells in the body, thereby assisting CTCs to evade the immune recognition (Wilson et al., 2010; Pieterse et al., 2017). 4) NETs can destroy the integrity of the vascular walls by adjusting the VE-cadherin and other ways to make tumor cells spill over, thus facilitating their metastasis to distant organs and the formation of micrometastases (Cools-Lartigue et al., 2014; Tohme et al., 2016). 5) NETs promote angiogenesis. Various components in NETs, such as: MMP-9 (Cools-Lartigue et al., 2013; Boone et al., 2015), CG (Cabel et al., 2017), and NE (Cools-Lartigue et al., 2013), can induce the expression of vascular endothelial growth factor (VEGF) to promote tumor growth, metastasis and angiogenesis. Under the stimulation of surgery, NETs can also stimulate Kupffer cells to release cytokines and chemokines, such as TNF-α (Moriai et al., 2009), IL-6 (Mcdonald et al., 2010) and CXCL-10 (Huh et al., 2010), and consequently induce tumor metastasis (Erpenbeck and Schön, 2017).

The use of inhibitors or degradation agents of NETs, such as DNase, for tumor treatment has been explored. Peptidyl arginine deminase4 (PAD4) can convert arginine to citrulline, which cause chromatin to decoagulate. Although stimulated by LPS and TNF, PAD4-deficient neutrophils cannot produce NETs (Zhou et al., 2018; Holmes et al., 2019; Galkina et al., 2020). NE inhibitors can also prevent the formation of NETs, handicap tumor cells to adhere to the capillary wall of liver or lung, and reduce tumor cell metastasis to the liver and lungs *in vivo* (Acuff et al., 2006). Whether NE and PAD4 inhibitors can affect tumor proliferation and metastasis by inhibiting the formation and release of NETs requires further verification.

## RCDs in Urinary Malignancies

### RCDs in Renal Malignancies

Renal cell carcinoma (RCC) is the most common type of kidney cancer. Although the primary lesion can be treated in surgery, approximately 40% of cases will recur and develop into metastatic tumor; the 5-years survival rate is 10% (Sánchez-Gastaldo et al., 2017).

Necroptosis has a double-faucet effect on the occurrence and development of malignant tumors, that is, it can promote and inhibit the biological process of malignant tumors. Most type of tumors have low RIPK3 expression and thus can resist necroptosis, which is conducive to tumor progression. (Sánchez-Gastaldo et al., 2017). However, high RIPK3 expression is also associated with poor patient survival in some type of tumors, such as ovarian (Colbert et al., 2013), colorectal (Zhai et al., 2016) and breast cancer (Li et al., 2017). Similar effects have also been found for MLKL expression (Nugues et al., 2014; Höckendorf et al., 2016; Liu et al., 2016; Zhai et al., 2016). RIPK1 and RIPK3 expression is significantly increased in high-grade clear cell renal carcinoma (ccRCC) *in vitro* compared with that in low-grade or normal cells (Cheng et al., 2021). Patients with papillary RCC and high miR-381-3p expression have a lower overall survival than those with low expression levels; miR-381-3p may blocks TNF-induced necroptosis by inhibiting the activation of RIPK3 and MLKL (Ou et al., 2018). Treating RCC cell lines (HK-2, ACHN, CaKi, 786-O, and OS-RC-2) with emodin can induce RIPK1 and MLKL phosphorylation, and result to necroptosis *in vitro* (Wilhelm et al., 2004). Artesunate can also inhibit the proliferation of kidney cancer cell lines *in vitro* by inducing ROS and thus increasing RIPK1-dependent necroptosis (Sonkusre and Cameotra, 2017).

Liver X receptor (LXR) is a member of the nuclear receptor superfamily mainly expressed in kidney (Wang Y. et al., 2017; Tan et al., 2020) and is one of the regulators of prostate (Katarina et al., 2017), breast (Hong and Tontonoz, 2008) and other types of cancer. In RCC, LXR-α can promote the metastasis by inhibiting NLRP3-inflammasome depended pyroptosis (Bobin-Dubigeon et al., 2017). Ursolic acid is a natural compound (Wang K. et al., 2019) that can up-regulate NLRP3 in RCC, then activate caspase-1, and eventually cause pyroptosis and inhibit tumor growth (Goodwin et al., 2008). On the contrary, resveratrol is a natural polyphenol compound that is widely present in crops such as peanuts and has anti-inflammatory effects (Chen Y.-M. et al., 2020). This compound substantially down-regulates NLRP3 while inhibiting RCC proliferation *in vitro* and can be blocked by NLRP3 inhibitors (Straif, 2009). Arsenic induces RCC by promoting AIM2-inflammasome and increasing the release of IL-1β and IL-18 to cause pyroptosis (Peng et al., 2020). These seemingly contradictory results also suggest that pyroptosis has a dual role in RCC.

The occurrence and development of tumors are often accompanied by redox imbalances and increased demand for iron ions. These phenomena suggest that tumor cells may have a higher sensitivity to ferroptosis (Weigand et al., 2020). In ccRCC, the research on ferroptosis is quite extensive. Low NCOA4 expression is related to the high TNM stage of ccRCC (Zou et al., 2019). Despite being a result of bio-information analysis based on TCGA and GEO databases, this finding points out potential research directions for follow-up clinical research. Therefore, other researchers have successively proposed prognostic survival models based on ferroptotic genes to clinically predict the survival and risk of patients with ccRCC (Markowitsch et al., 2020; Wu et al., 2020). The prognostic characteristics of ferroptotic genes, such as NCOA4 and SLC7A11, were found to be independent prognostic factors for patients with ccRCC. Subsequent studies showed that ccRCC depends on GPX4 to reduce lipid peroxides (Chang et al., 2021) and extremely sensitive to the lack of glutamine and cystine. If the synthesis of GSH is inhibited, then ferroptosis can be induced in ccRCC to limit tumor growth (Mou et al., 2021). In addition, ferroptosis may also be a potential target to resolve drug resistance in RCC. Artesunate can inhibit the growth of sunitinib-resistant RCC by inducing ferroptosis (Miess et al., 2018). A high ferroptotic score indicates poor ccRCC prognosis but high sensitivity to chemotherapy drugs such as vinorelbine (Bai et al., 2021).

CTCs are a main factor that cause RCC metastasis. The grid-like structure of NETs and the tissue factor (TF) can form tangles with CTCs. Therefore, the scores of NETs and TF were identified as independent risk factors for patients of RCC (Guan et al., 2015). Inhibiting MMP-9 can also suppress RCC proliferation and metastasis *in vitro* (Guan et al., 2015; Mego et al., 2019). Conversely, promoting MMP-9 can enhance the proliferation and migration of tumor (Mego et al., 2019). MMP-9 is also a necessary substance in ccRCC to promote angiogenesis (Guo et al., 2019).

### RCDs in Bladder Malignancies

Bladder cancer is the 4th most common and 8th most lethal malignancy among men in the United States (Siegel et al., 2020). ABT-737 is an inhibitor of Bcl-2 and inhibits its biological activity by competitively binding Bcl-2 (Wang et al., 2021). In bladder cancer, ABT-737 can directly induce MLKL-mediated necroptosis by upregulating RIPK3 expression without requiring RIPK1 (Haigis and Sinclair, 2010). However, only a few studies were conducted on necroptosis in bladder cancer. Hence, many unknown mechanisms remain in this area of research and must be explored.

High GSDMD expression promotes the proliferation of bladder cancer cells and is related to the reduced in overall survival (Poli et al., 2020). This phenomenon may be related to the inflammatory environment caused by severe pyroptosis and provides suitable conditions for the proliferation of bladder cancer. However, NLRP3, NLRP4, and NLRP9 were significantly increased in the urine and tumor tissue samples of patients with bladder cancer compared with those in healthy people (Poli et al., 2020). Different inflammasomes (NLRP1, NLRP2, NLRP3, NLRP4, NLRP5, NLRP6, NLRP7, NLRP12 and AIM2) determine the difference of pathological features in bladder cancer (Tian et al., 2020). Therefore, studies on pyroptosis in bladder cancer may require individualized research plans and evaluation indicators. These individualized differences can further help physicians to accurately determine patient prognosis.

Quinazolinyl-arylurea derivative named 7j was obtained by modifying the chemical structure of sorafenib (Ceballos et al., 2011). 7j can cause bladder cancer cells death by regulating the xCT/GPX4/ROS pathway *in vitro* and exhibits suitable affinity for GPX4 (Ceballos et al., 2011). MiRNA-27a down-regulation can targetedly promote SLC7A11 and regulate GSH biosynthesis, thus enhancing the resistance of bladder cancer cells to cisplatin *in vitro* (Qin et al., 2021).

BCG perfusion has been used a treatment of bladder cancer (Saluja and Gilling, 2017) and still occupies an important position in anti-bladder cancer therapeutics (Gontero et al., 2010; Lin et al., 2015). BCG was recently discovered to possible inhibit the proliferation of bladder cancer by inducing the NETs production. The mechanism may be related to tumor damage and the enhancement of T cells and monocyte-macrophages in TME by NETs (Gontero et al., 2010; Lin et al., 2015). Studies on whether NETs can directly inhibit tumor growth are currently lacking. Hence, their potential anti-tumor effects as a part of innate immunity remain to be discovered. Nevertheless, NETs or the associated components remain to be poor prognostic factors in bladder cancer. For example, NETs can promote bladder cancer resist to radiotherapy (Wu et al., 2018); MMP-9 is significantly expressed in high-grade bladder cancer compare with that in low-grade ones (Shinde-Jadhav et al., 2021). The high expression of MMP-9 can promote the metastasis of bladder cancer has been confirmed, and there is evidence that the process is associated with MMP-9 promoted epithelial-mesenchymal transition (EMT) in bladder cancer (Ashrafizadeh et al., 2020a; Mirzaei et al., 2021a; Mirzaei et al., 2021b). NE can promote the proliferation and invasion of bladder cancer and other malignant behaviors *in vitro* (Yang and Sun, 2004). Balancing the anti-tumor effect and tumor-promoting effect of NETs in bladder cancer is clinically significant and challenging task.

### RCDs in Prostate Malignancies

Prostate cancer is the most prevalent malignancy among men in the United States, ranking the 2nd in the mortality rate of malignant tumors (Siegel et al., 2020).

RIPK3 expression is significantly decreased in prostate cancer compared with that in normal tissues, and its overexpression significantly inhibits the proliferation and invasion of prostate cancer *in vitro* and *in vivo*. For example, RIPK3 inhibits prostate cancer progression by activating MLKL through phosphorylation and necroptosis activation (Zhao et al., 2020). In advanced prostate cancer, RIPK3 is significantly suppressed at a rate proportional to tumor size and prostate-specific antigen (PSA) (Lu et al., 2020). Sirtuin (SIRT) family is consisted of seven members (SIRT1-7) (Michan and Sinclair, 2007; Chauhan et al., 2017). Although they all share a conserved catalytic core structural domain, their different enzymatic activities and cellular localization result in diverse functions; the members are involved in various biological behaviors including cellular metabolism, DNA repair, tumor development, and cellular senescence (Marmorstein, 2004). In prostate cancer, SIRT-3 and SIRT-6 can protect prostate cancer cells from necroptosis and reduce the overall survival (Fu et al., 2020). Sorafenib is a 2nd generation tyrosine kinase inhibitor (TKI) that targets Raf kinases, including Raf-1 and b-Raf, VEGFR-2 and -3, PDGFR-β, Flt-3, and c-KIT (Kharaziha et al., 2015). In the Atg5-deficient prostate cancer cell line DU-145, sorafenib could mediate necroptosis by inducing the RIPK1/RIPK3/MLKL pathway, and this process can be blocked by the RIPK1 inhibitor Nec-1 (Heidaryan et al., 2020). Even in androgen-dependent prostate cancer, necroptosis can be induced in LNCaP cells through activation of RIPK1 by Ophiopogonin D′ (OPD′); the use of either Nec-1 or necrosulfonamide, a kind of MLKL inhibitor, can inhibit necroptosis induced by OPD′ (Wang J. et al., 2017). In summary, necroptosis shows promise as a new target for the treatment of prostate cancer. Therefore, some investigators have developed a novel bio-selenium nanoparticle in prostate cancer that can cause necroptosis by inducing TNF-α activation of RIPK1 (Al-Lamki et al., 2016).

Prostate cancer and its paracancerous tissues usually infiltrated a large number of inflammatory cells. The inflammatory microenvironment may be an important factor for promoting tumor cells growth (Khan et al., 2016). On the one hand, it recruits the inflammatory cells and releases the factors to promote tumor growth. On the other hand, it activates TGF-β and inhibits the expression of caspase-1 and the maturation and release of IL-1β (Khan et al., 2016). NLRP12 has similar functions to TGF-β and can also promote the occurrence and development of prostate cancer by regulating caspase-1 and its downstream IL-1β and IL-18 (Karan et al., 2017). Caspase-3 could be activated by chemotherapeutic or targeted drugs and cutting GSDME in the prostate cancer tissues to cause pyroptosis provide anti-tumor effects (Hersh et al., 1999). Therefore, caspase-3 could become a target in prostate cancer treatment.

The beta-oxidation of fatty acids is the most important energy metabolism pathway in prostate cancer (Liu, 2006; Balaban et al., 2019). DECR1 can cause PUFA depletion in prostate cancer by encoding the rate-limiting enzyme of PUFA oxidation, thus protecting prostate cancer from ferroptosis. Targeting to DECR1 can cause PUFA to accumulate in cells, enhanced oxidative stress and lipids peroxidation, and finally induce ferroptosis (Nassar et al., 2020; Yang et al., 2021). Erastin and RSL3, showing a good ability to induce ferroptosis in prostate cancer *in vitro* and suppress this malignancy (Yang et al., 2021; Ghoochani A et al., 2021). Erastin can down-regulate the androgen receptor (AR) in castration-resistant prostate cancer (CRPC) and thus have potential anti-CRPC effects (Ghoochani et al., 2021). As a great clinical challenge, CRPC is expected to be treated by ferroptosis. Some researchers synthesized a hybrid AR antagonist (ITC-ARi) containing isothiocyanate, which can down-regulate AR and enhance the sensitivity of CRPC cells to ferroptosis inducers such as erasitin (Yang et al., 2021). Flubendazole is a broad-spectrum antiparasitic drug (Ceballos et al., 2009; Mackenzie and Geary, 2011; Zhou et al., 2020) that can induce the P53 expression in CRPC cells, inhibit SLC7A11 transcription, and down-regulating GPX4 to promote ferroptosis (Chen J.-N. et al., 2020). In summary, prostate cancer cells are highly sensitive to ferroptosis due to their unique androgen dependence and energy metabolism characteristics. So, ferroptosis may have a good therapeutic potential. In-depth basic research and clinical cohort analysis with large sample size are still needed to evaluate the effectiveness of this treatment.

NETs components possibly cause the proliferation and migration of prostate cancer cells. For example, NE can promote the proliferation of prostate cancer *in vitro* (Nakazawa et al., 2012; Lerman et al., 2019). The risk of venous thromboembolism (VTE) in prostate cancer is significantly high (Nakazawa et al., 2012; Lerman et al., 2019), and NETs can mediate the formation of clots in patients with malignancies through various ways. NETs are also found in human and animal thrombosis. The knocked down the PAD4 gene in mice indicated that NETs are a component of venous thrombosis involved in the formation and maintenance of thrombus (Longstaff et al., 2013; Martinod et al., 2013; Ording et al., 2015). Some researchers believe that this is because microparticles (MPs) can combine with TF, CG and other substances on the surface of NETs to promote blood clotting and form thrombus (Thomas et al., 2015).

## RCDs in Anti-Tumor Agents

With the improvement of research on RCD, RCD regulated drugs in tumor therapy have been gaining attention.

Chemotherapy is the longest use and the widest coverage in tumor therapy. The main mechanism is to inhibit cell proliferation and promote cell death. In the past, most of the chemotherapeutic drugs used to promote cell apoptosis to limit the development of tumors. However, accumulated evidence shows that promoting necroptosis or ferroptosis maybe achieve better therapeutic effecacy. Artemisinin (ART) is used to treat malaria and has been approved by the FDA for the treatment of cancer. ART and its derivatives induce ferroptosis by promoting degradation of ferritin in lysosomal, down-regulating the expression of GPX4 and inducing the production of lipid peroxide (Ou et al., 2017; Chen et al., 2019).

With the understanding of cancer molecular biology continues advance, drug development has shifted towards agents that target specific molecular alternations in tumors. These molecular targeted therapies have achieved varying degrees of success. Sorafenib can not only induce necroptosis in prostate cancer, but also has been reported to induce ferroptosis in liver cancer (Wilhelm et al., 2004; Longstaff et al., 2013; Louandre et al., 2013; Dixon et al., 2014). A novel type of chalcone was discovered by inducing caspase-3 and then activating GSDME to induce pyroptosis for the treatment of CRPC. Inducing cancer cells to produce pyroptosis can effectively inhibit tumor growth, but the inflammatory microenvironment can cause tumor cell proliferation. Therefore, maximizing pyroptosis in prostate cancer while limited the scale and scope of inflammation could be a focal point in future. For example, a drug which targets and activates NLRP3 can suppress IL-1β and IL-18 while causing prostate cancer pyroptosis and then limiting the scale and scope of inflammation (Dixon et al., 2012; Li et al., 2020).

RCD can also play an important role in immunotherapy. For example: interferon-gamma (IFNγ) released by CD8+ T cells can down-regulate the expression of SLC3A2 and SLC7A1 in tumor cells, inhibiting the uptake of cystine, limiting tumor development and improving patient prognosis. T cells promote tumor ferroptosis is a new mechanism of tumor immunotherapy, and combining it with immune checkpoints may become a new strategy for anti-tumor therapy (Wang W. et al., 2019).

Inhibiting tumor growth by inducing RCD will surely become a new opportunity for drug design in the future. However, there are many obstacles to the use of these agents in clinical treatment. For example: agents that induce pyroptosis will inevitably cause the release of inflammatory factors, and the followed cytokine storm may endanger the lives of patients. Secondly, targeting tumor cells is also a obstacle that must be solved before clinical application. Nano-vehicles agents may be a solution.

Nano-vehicles are being used to the development of anti-tumor agents because of they have advantages in stability, degradability, targeted delivery, high bioavailability and less side effects. In prostate cancer, nano-vehicles drugs have been widely studied and used (Ashrafizadeh et al., 2020b; Hussain et al., 2021). Nano-vehicles agents to induce ferroptosis in tumor cells is a fresh and potential direction of new drug design. PUFA can effectively prevent tumors through ferroptosis. However, the therapeutic effects of these lipids on tumors are not optimistic once tumors are formed. A kind of nanoparticles constructed with low-density lipoprotein (LDL) and PUFA can directly inhibit the growth of transplanted tumors in mice. PEGylated single-atom Fe-containing nanocatalysts (PSAF NCs) is designed by nanoparticulate drug delivery systems (nano-DDS) which containing iron atoms and polyethylene glycol (PEG). It can disperse iron atoms into carbon nano-materials, and catalyzes specifically the Fenton reaction. According to the good ability of degradation and biocompatibility of the nanomaterial, it has great potential to be used in clinical anti-tumor therapy (Sawada et al., 2012; Ou et al., 2017; Huo et al., 2019).

## Conclusion

Cell death is the process by which living cells stop functioning and can happen in many ways for different reasons. Cells may die due to cellular stress and metabolic destruction, pathogen invasion, or damage to physiological tissues during development. With further research, the regulation mechanism of RCD and its mode of action in tumors have been clearly explained.

RCD is not only a pathological phenomenon, but also should be used in the evaluation and treatment of malignancies. Tumor is currently the most serious disease endangering people’s health, and the economic and medical burden it brings is huge for every country. However, there are few drugs for the treatment in tumors through RCD at present, and the research mainly focuses on the areas of ferroptosis and necroptosis, while drugs for the treatment of tumors through NETs are very rare. It has great potential to treat malignant tumors through the interaction between NETs and TME (Chernyak et al., 2021). BCG therapy in bladder cancer is a good example. Therefore we hope more attention to be payed in this area.

This work reviewed the mechanism of necroptosis, pyroptosis, ferroptosis, and NETs and the relationship between the occurrence and regulation in urinary malignancies. Many components of RCD pathways can serve as key biomarkers and potential therapeutic targets. These key protein targets in cell death pathways provide broad prospects for translational research and the treatment of various malignant tumors.

RCD is a developing concept. Not only are the molecular mechanisms of these death modes poorly understood, but new RCD modes are constantly being proposed. PANoptosis (Py-, Ap-, Necr-optosis) is a unique inflammatory RCD mode that regulated by PANoptosome. Early studies found that pyroptosis can cleave other caspase and PARP1 through caspase-1, and apoptotic caspase-8 is very important for NLRP3-dependent inflammasomes. Because this mode of RCD has the complex characteristics of pyroptosis, apoptosis and necroptosis, it is named PANoptosis (Karki et al., 2020; Place et al., 2021). There are few studies in PANoptosis and tumors, but a study have reported that IRF1-dependent PANoptosis activation can prevent colorectal tumors in mouse, suggesting that PANoptosis plays a broader role in cancer (Karki et al., 2020; Place et al., 2021). This shows that research on RCD will definitely improve the prognosis of clinical patients.

## References

[B1] Acuff H. B. Carter K. J. Fingleton B. Gorden D. L. Matrisian L. M. (2006). Matrix Metalloproteinase-9 from Bone Marrow-Derived Cells Contributes to Survival but Not Growth of Tumor Cells in the Lung Microenvironment. Cancer Res. 66 (1), 259–266. 10.1158/0008-5472.CAN-05-2502 16397239 PMC1360653

[B2] Aglietti R. A. Estevez A. Gupta A. Ramirez M. G. Liu P. S. Kayagaki N. (2016). GsdmD P30 Elicited by Caspase-11 during Pyroptosis Forms Pores in Membranes. Proc. Natl. Acad. Sci. USA 113 (28), 7858–7863. 10.1073/pnas.1607769113 27339137 PMC4948338

[B3] Al-Lamki R. S. Lu W. Manalo P. Wang J. Warren A. Y. Tolkovsky A. M. (2016). Tubular Epithelial Cells in Renal clear Cell Carcinoma Express High RIPK1/3 and Show Increased Susceptibility to TNF Receptor 1-induced Necroptosis. Cell Death Dis. 7 (6), e2287. 10.1038/cddis.2016.184 27362805 PMC5108336

[B4] Amulic B. Hayes G. (2011). Neutrophil Extracellular Traps. Curr. Biol. 21 (9), R297–R298. 10.1016/j.cub.2011.03.021 21549944

[B5] Amulic B. Knackstedt S. L. Abu Abed U. Deigendesch N. Harbort C. J. Caffrey B. E. (2017). Cell-Cycle Proteins Control Production of Neutrophil Extracellular Traps. Dev. Cel. 43 (4), 449–462. 10.1016/j.devcel.2017.10.013 29103955

[B6] Ashrafizadeh M. Hushmandi K. Hashemi M. Akbari M. E. Kubatka P. Raei M. (2020a). Role of microRNA/Epithelial-To-Mesenchymal Transition Axis in the Metastasis of Bladder Cancer. Biomolecules 10 (8), 1159. 10.3390/biom10081159 PMC7464913 32784711

[B7] Ashrafizadeh M. Hushmandi K. Moghadam E. R. Zarrin V. Kashani S. H. Bokaie S. (2020b). Progress in Delivery of siRNA-Based Therapeutics Employing Nano-Vehicles for Treatment of Prostate Cancer. Bioengineering (Basel) 7 (3), 91. 10.3390/bioengineering7030091 PMC7552721 32784981

[B8] Bai D. Feng H. Yang J. Yin A. Sugiyama H. (2021). Genomic Analysis Uncovers Prognostic and Immunogenic Characteristics of Ferroptosis for clear Cell Renal Cell Carcinoma. Mol. Ther. - Nucleic Acids 25, 186–197. 10.3390/bioengineering7030091 34458004 PMC8368772

[B9] Balaban S. Nassar Z. D. Zhang A. Y. Hosseini-Beheshti E. Centenera M. M. Schreuder M. (2019). Extracellular Fatty Acids Are the Major Contributor to Lipid Synthesis in Prostate Cancer. Mol. Cancer Res. 17 (4), 949–962. 10.1158/1541-7786.MCR-18-0347 30647103

[B10] Bersuker K. Hendricks J. M. Li Z. Magtanong L. Ford B. Tang P. H. (2019). The CoQ Oxidoreductase FSP1 Acts Parallel to GPX4 to Inhibit Ferroptosis. Nature 575 (7784), 688–692. 10.1038/s41586-019-1705-2 31634900 PMC6883167

[B11] Blackwell K. Zhang L. Workman L. M. Ting A. T. Iwai K. Habelhah H. (2013). Two Coordinated Mechanisms Underlie Tumor Necrosis Factor Alpha-Induced Immediate and Delayed IκB Kinase Activation. Mol. Cel Biol. 33 (10), 1901–1915. 10.1128/mcb.01416-12 PMC3647962 23459942

[B12] Bobin-Dubigeon C. Chauvin A. Brillaud-Meflah V. Boiffard F. Joalland M. P. Bard J. M. (2017). Liver X Receptor (LXR)-regulated Genes of Cholesterol Trafficking and Breast Cancer Severity. Anticancer Res. 37 (10), 5495–5498. 10.21873/anticanres.11979 28982861

[B13] Boone B. A. Orlichenko L. Schapiro N. E. Loughran P. Gianfrate G. C. Ellis J. T. (2015). The Receptor for Advanced Glycation End Products (RAGE) Enhances Autophagy and Neutrophil Extracellular Traps in Pancreatic Cancer. Cancer Gene Ther. 22 (6), 326–334. 10.1038/cgt.2015.21 25908451 PMC4470814

[B14] Brault M. Olsen T. M. Martinez J. Stetson D. B. Oberst A. (2018). Intracellular Nucleic Acid Sensing Triggers Necroptosis through Synergistic Type I IFN and TNF Signaling. J. Immunol. 200 (8), 2748–2756. 10.4049/jimmunol.1701492 29540580 PMC5893403

[B15] Bridges R. J. Natale N. R. Patel S. A. (2011). System Xc⁻ Cystine/glutamate Antiporter: an Update on Molecular Pharmacology and Roles within the CNS. Br. J. Pharmacol. 165, 20–34. 10.1111/j.1476-5381.2011.01480.x PMC3252963 21564084

[B16] Brinkmann V. Reichard U. Goosmann C. Fauler B. Uhlemann Y. Weiss D. S. (2004). Neutrophil Extracellular Traps Kill Bacteria. Science 303 (5663), 1532–1535. 10.1126/science.1092385 15001782

[B17] Cabel L. Proudhon C. Gortais H. Loirat D. Coussy F. Pierga J. Y. (2017). Circulating Tumor Cells: Clinical Validity and Utility. Int. J. Clin. Oncol. 22 (3), 421–430. 10.1007/s10147-017-1105-2 28238187

[B18] Cai Z. Jitkaew S. Zhao J. Chiang H.-C. Choksi S. Liu J. (2014). Plasma Membrane Translocation of Trimerized MLKL Protein Is Required for TNF-Induced Necroptosis. Nat. Cel Biol. 16 (1), 55–65. 10.1038/ncb2883 PMC8369836 24316671

[B19] Castanheira F. V. S. Kubes P. (2019). Neutrophils and NETs in Modulating Acute and Chronic Inflammation. Blood 133 (20), 2178–2185. 10.1182/blood-2018-11-844530 30898862

[B20] Ceballos L. Elissondo M. Bruni S. S. Denegri G. Alvarez L. Lanusse C. (2009). Flubendazole in Cystic Echinococcosis Therapy: Pharmaco-Parasitological Evaluation in Mice. Parasitol. Int. 58 (4), 354–358. 10.1016/j.parint.2009.07.006 19628054

[B21] Ceballos L. Elissondo C. Sánchez Bruni S. Denegri G. Lanusse C. Alvarez L. (2011). Comparative Performances of Flubendazole and Albendazole in Cystic Echinococcosis:Ex VivoActivity, Plasma/Cyst Disposition, and Efficacy in Infected Mice. Antimicrob. Agents Chemother. 55 (12), 5861–5867. 10.1128/aac.05105-11 21930885 PMC3232756

[B22] Chan F. (2015). Programmed Necrosis in the Cross Talk of Cell Death and Inflammation. Annu. Rev. Immunol. 33, 79–106. 10.1146/annurev-immunol-032414-112248 25493335 PMC4394030

[B23] Chang K. Yuan C. Liu X. (2021). Ferroptosis-Related Gene Signature Accurately Predicts Survival Outcomes in Patients with Clear-Cell Renal Cell Carcinoma. Front. Oncol. 11, 649347. 10.3389/fonc.2021.649347 33996565 PMC8120155

[B24] Chauhan A. K. Min K. J. Kwon T. K. (2017). RIP1-dependent Reactive Oxygen Species Production Executes Artesunate-Induced Cell Death in Renal Carcinoma Caki Cells. Mol. Cell Biochem. 435 (1-2), 15–24. 10.1007/s11010-017-3052-7 28466458

[B25] Chen X. Li W. Ren J. Huang D. He W. T. Song Y. (2014). Translocation of Mixed Lineage Kinase Domain-like Protein to Plasma Membrane Leads to Necrotic Cell Death. Cell Res. 24 (001), 105–121. 10.1038/cr.2013.171 24366341 PMC3879712

[B26] Chen W. Zheng R. Baade P. D. Zhang S. Zeng H. Bray F. (2016). Cancer Statistics in China, 2015. CA: a Cancer J. Clin. 66 (2), 115–132. 10.3322/caac.21338 26808342

[B27] Chen G. Q. Benthani F. A. Wu J. Liang D. Bian Z. X. Jiang X. (2019). Artemisinin Compounds Sensitize Cancer Cells to Ferroptosis by Regulating Iron Homeostasis. Cell Death Differ. 27 (Suppl. 1), 242–254. 10.1038/s41418-019-0352-3 31114026 PMC7205875

[B28] Chen J.-N. Li T. Cheng L. Qin T.-S. Sun Y.-X. Chen C.-T. (2020a). Synthesis and *In Vitro* Anti-bladder Cancer Activity Evaluation of Quinazolinyl-Arylurea Derivatives. Eur. J. Med. Chem. 205, 112661. 10.1016/j.ejmech.2020.112661 32827851

[B29] Chen Y.-M. Tang B.-X. Chen W.-Y. Zhao M.-S. (2020b). Ursolic Acid Inhibits the Invasiveness of A498 Cells via NLRP3 Inflammasome Activation. Oncol. Lett. 20 (5), 170. 10.3892/ol.2020.12027 32934737 PMC7471750

[B30] Cheng R. Liu X. Wang Z. Tang K. (2021). ABT-737, a Bcl-2 Family Inhibitor, Has a Synergistic Effect with Apoptosis by Inducing Urothelial Carcinoma Cell Necroptosis. Mol. Med. Rep. 23 (6), 412. 10.3892/mmr.2021.12051 33786632 PMC8025475

[B31] Chernyak B. V. Sokolov A. V. Hwang T. L. Zinovkin R. A. Sud'Ina G. F. (2021). Editorial: Pharmacological Approaches Targeting Neutrophilic Inflammation. Front. Pharmacol. 12, 763140. 10.3389/fphar.2021.763140 34588989 PMC8473688

[B32] Colbert L. E. Fisher S. B. Hardy C. W. Hall W. A. Saka B. Shelton J. W. (2013). Pronecrotic Mixed Lineage Kinase Domain-Like Protein Expression Is a Prognostic Biomarker in Patients with Early-Stage Resected Pancreatic Adenocarcinoma. Cancer 119 (17), 3148–3155. 10.1002/cncr.28144 23720157 PMC4389890

[B33] Colotta F. Allavena P. Sica A. Garlanda C. Mantovani A. (2009). Cancer-related Inflammation, the Seventh Hallmark of Cancer: Links to Genetic Instability. Carcinogenesis 30 (7), 1073–1081. 10.1093/carcin/bgp127 19468060

[B34] Cookson B. T. Brennan M. A. (2001). Pro-inflammatory Programmed Cell Death. Trends Microbiol. 9 (3), 113–114. 10.1016/s0966-842x(00)01936-3 11303500

[B35] Cools-Lartigue J. Spicer J. Mcdonald B. Gowing S. Chow S. Giannias B. (2013). Neutrophil Extracellular Traps Sequester Circulating Tumor Cells and Promote Metastasis. J. Clin. Invest. 123 (8), 3446–3458. 10.1172/JCI67484 PMC3726160 23863628

[B36] Cools-Lartigue J. Spicer J. Najmeh S. Ferri L. (2014). Neutrophil Extracellular Traps in Cancer Progression. Cell. Mol. Life Sci. 71 (21), 4179–4194. 10.1007/s00018-014-1683-3 25070012 PMC7096049

[B37] D’ Herde K. Krysko D. V. (2016). Ferroptosis: Oxidized PEs Trigger Death. Nat. Chem. Biol. 13 (1), 4–5. 10.1038/nchembio.2261 27842067

[B38] Degterev A. Hitomi J. Germscheid M. Ch'en I. L. Korkina O. Teng X. (2008). Identification of RIP1 Kinase as a Specific Cellular Target of Necrostatins. Nat. Chem. Biol. 4 (5), 313–321. 10.1038/nchembio.83 18408713 PMC5434866

[B39] Demers M. Wong S. L. Martinod K. Gallant M. Cabral J. E. Wang Y. (2016). Priming of Neutrophils toward NETosis Promotes Tumor Growth. Oncoimmunology 5 (5), e1134073. 10.1080/2162402X.2015.1134073 27467952 PMC4910712

[B40] Dillon C. P. Oberst A. Weinlich R. Janke L. J. Kang T.-B. Ben-Moshe T. (2012). Survival Function of the FADD-CASPASE-8-cFLIPL Complex. Cel Rep. 1 (5), 401–407. 10.1016/j.celrep.2012.03.010 PMC3366463 22675671

[B41] Dinarello C. A. (2009). Immunological and Inflammatory Functions of the Interleukin-1 Family. Annu. Rev. Immunol. 27 (1), 519–550. cytokine, host defense, caspase-1, autoinflammatory, inflammasome. 10.1146/annurev.immunol.021908.132612 19302047

[B42] Ding J. Wang K. Liu W. She Y. Sun Q. Shi J. (2016). Pore-forming Activity and Structural Autoinhibition of the Gasdermin Family. Nature 535 (7610), 111–116. 10.1038/nature18590 27281216

[B43] Dixon S., J. Lemberg K., M. Lamprecht M., R. Skouta R. Zaitsev E. M. Gleason C. E. (2012). Ferroptosis: An Iron-dependent Form of Nonapoptotic Cell Death. Cell 149 (5), 1060–1072. 10.1016/j.cell.2012.03.042 22632970 PMC3367386

[B44] Dixon S. J. Patel D. N. Welsch M. Skouta R. Stockwell B. R. (2014). Pharmacological Inhibition of Cystine-Glutamate Exchange Induces Endoplasmic Reticulum Stress and Ferroptosis. eLife Sci. 3, e02523. 10.7554/eLife.02523 PMC4054777 24844246

[B45] Doll S. Freitas F. P. Shah R. Aldrovandi M. da Silva M. C. Ingold I. (2019). FSP1 Is a Glutathione-independent Ferroptosis Suppressor. Nature 575 (7784), 693–698. 10.1038/s41586-019-1707-0 31634899

[B46] Dolma S. Lessnick S. L. Hahn W. C. Stockwell B. R. (2003). Identification of Genotype-Selective Antitumor Agents Using Synthetic Lethal Chemical Screening in Engineered Human Tumor Cells. Cancer Cel. 3 (3), 285–296. 10.1016/s1535-6108(03)00050-3 12676586

[B47] Dondelinger Y. Declercq W. Montessuit S. Roelandt R. Goncalves A. Bruggeman I. (2014). MLKL Compromises Plasma Membrane Integrity by Binding to Phosphatidylinositol Phosphates. Cel. Rep. 7 (4), 971–981. 10.1016/j.celrep.2014.04.026 24813885

[B48] Dondelinger Y. Darding M. Bertrand M. J. M. Walczak H. (2016). Poly-ubiquitination in TNFR1-Mediated Necroptosis. Cel. Mol. Life Sci. 73, 2165–2176. 10.1007/s00018-016-2191-4 PMC4887548 27066894

[B49] Erpenbeck L. Schön M. P. (2017). Neutrophil Extracellular Traps: Protagonists of Cancer Progression? Oncogene 36 (18), 2483–2490. 10.1038/onc.2016.406 27941879

[B50] Fu W. Li H. Fu H. Zhao S. Shi W. Sun M. (2020). The SIRT3 and SIRT6 Promote Prostate Cancer Progression by Inhibiting Necroptosis-Mediated Innate Immune Response. J. Immunol. Res. 2020 (1), 1–12. 10.1155/2020/8820355 PMC7685829 33282964

[B51] Fuchs T. A. Abed U. Goosmann C. Hurwitz R. Schulze I. Wahn V. (2007). Novel Cell Death Program Leads to Neutrophil Extracellular Traps. J. Cel Biol. 176 (2), 231–241. 10.1083/jcb.200606027 PMC2063942 17210947

[B52] Galkina S. I. Fedorova N. V. Golenkina E. A. Stadnichuk V. I. Sud'Ina G. F. (2020). Cytonemes versus Neutrophil Extracellular Traps in the Fight of Neutrophils with Microbes. Int. J. Mol. Sci. 21 (2), 586. 10.3390/ijms21020586 PMC7014225 31963289

[B53] Galluzzi L. Kepp O. Chan F. K.-M. Kroemer G. (2017). Necroptosis: Mechanisms and Relevance to Disease. Annu. Rev. Pathol. Mech. Dis. 12, 103–130. 10.1146/annurev-pathol-052016-100247 PMC5786374 27959630

[B54] Galluzzi L. Vitale I. Aaronson S. A. Abrams J. M. Kroemer G. (2018). Molecular Mechanisms of Cell Death: Recommendations of the Nomenclature Committee on Cell Death 2018. Cel Death Differ. 25, 486–541. 10.1038/s41418-017-0012-4 PMC5864239 29362479

[B55] Gao M. Monian P. Quadri N. Ramasamy R. Jiang X. (2015). Glutaminolysis and Transferrin Regulate Ferroptosis. Mol. Cel. 59 (2), 298–308. 10.1016/j.molcel.2015.06.011 PMC4506736 26166707

[B56] Gaschler M. M. Stockwell B. R. (2017). Lipid Peroxidation in Cell Death. Biochem. Biophys. Res. Commun. 482 (3), 419–425. 10.1016/j.bbrc.2016.10.086 28212725 PMC5319403

[B57] Ghoochani A. Hsu E.-C. Aslan M. Rice M. A. Nguyen H. M. Brooks J. D. (2021). Ferroptosis Inducers Are a Novel Therapeutic Approach for Advanced Prostate Cancer. Cancer Res. 81 (6), 1583–1594. 10.1158/0008-5472.Can-20-3477 33483372 PMC7969452

[B58] Gontero P. Bohle A. Malmstrom P.-U. O’Donnell M. A. Oderda M. Sylvester R. (2010). The Role of Bacillus Calmette-Guérin in the Treatment of Non-Muscle-Invasive Bladder Cancer. Eur. Urol. 57 (3), 410–429. 10.1016/j.eururo.2009.11.023 19969411

[B59] Goodwin B. J. Zuercher W. J. Collins J. L. (2008). Recent Advances in Liver X Receptor Biology and Chemistry. Curr. Top. Med. Chem. 8 (9), 781–791. 10.2174/156802608784535075 18537688

[B60] Grootjans S. Berghe T. V. Vandenabeele P. (2017). Initiation and Execution Mechanisms of Necroptosis: an Overview. Cel Death Differ. 24 (7), 1184–1195. 10.1038/cdd.2017.65 PMC5520172 28498367

[B61] Guan B. Z. Yan R. L. Huang J. W. Li F. L. Zhong Y. X. Chen Y. (2015). Activation of G Protein Coupled Estrogen Receptor (GPER) Promotes the Migration of Renal Cell Carcinoma via the PI3K/AKT/MMP-9 Signals. Cell Adh Migr. 12 (2), 109–117. 10.4161/19336918.2014.990781 PMC5927669 25588050

[B62] Guo Y. Xu F. Lu T. Duan Z. Zhang Z. (2012). Interleukin-6 Signaling Pathway in Targeted Therapy for Cancer. Cancer Treat. Rev. 38 (7), 904–910. 10.1016/j.ctrv.2012.04.007 22651903

[B63] Guo F. Liu J. Han X. Zhang X. Lin T. Wang Y. (2019). FBXO22 Suppresses Metastasis in Human Renal Cell Carcinoma via Inhibiting MMP-9-Mediated Migration and Invasion and VEGF-Mediated Angiogenesis. Int. J. Biol. Sci. 15 (3), 647–656. 10.7150/ijbs.31293 30745851 PMC6367582

[B64] Haigis M. C. Sinclair D. A. (2010). Mammalian Sirtuins: Biological Insights and Disease Relevance. Annu. Rev. Pathol. Mech. Dis. 5, 253–295. 10.1146/annurev.pathol.4.110807.092250 PMC2866163 20078221

[B65] Hayden M. S. Ghosh S. (2014). Regulation of NF-κB by TNF Family Cytokines. Semin. Immunol. 26 (3), 253–266. 10.1016/j.smim.2014.05.004 24958609 PMC4156877

[B66] Heidaryan F. Bamehr H. Babaabasi B. Emamvirdizadeh A. Mohammadzadeh N. Khalili A. (2020). The Trend of Ripk1/ripk3 and Mlkl Mediated Necroptosis Pathway in Patients with Different Stages of Prostate Cancer as Promising Progression Biomarkers. Clin. Lab. 66 (3). 10.7754/Clin.Lab.2019.190439 32162861

[B67] Hentze M. W. Muckenthaler M. U. Galy B. Camaschella C. (2010). Two to Tango: Regulation of Mammalian Iron Metabolism. Cell 142 (1), 24–38. 10.1016/j.cell.2010.06.028 20603012

[B68] Hersh D. Monack D. M. Smith M. R. Ghori N. Falkow S. Zychlinsky A. (1999). The Salmonella Invasin SipB Induces Macrophage Apoptosis by Binding to Caspase-1. Proc. Natl. Acad. Sci. 96 (5), 2396–2401. 10.1073/pnas.96.5.2396 10051653 PMC26795

[B69] Hilbi H. Chen Y. Thirumalai K. Zychlinsky A. (1997). The Interleukin 1beta-Converting Enzyme, Caspase 1, Is Activated during Shigella Flexneri-Induced Apoptosis in Human Monocyte-Derived Macrophages. Infect. Immun. 65 (12), 5165–5170. 10.1128/iai.65.12.5165-5170.1997 9393811 PMC175744

[B70] Hitomi J. Christofferson D. E. Ng A. Yao J. Degterev A. Xavier R. J. (2008). Identification of a Molecular Signaling Network that Regulates a Cellular Necrotic Cell Death Pathway. Cell 135 (7), 1311–1323. 10.1016/j.cell.2008.10.044 19109899 PMC2621059

[B71] Höckendorf U. Yabal M. Herold T. Munkhbaatar E. Rott S. Jilg S. (2016). RIPK3 Restricts Myeloid Leukemogenesis by Promoting Cell Death and Differentiation of Leukemia Initiating Cells. Cancer Cell 30 (1), 75–91. 10.1016/j.ccell.2016.06.002 27411587

[B72] Holmes C. L. Shim D. Kernien J. Johnson C. J. Nett J. E. Shelef M. A. (2019). Insight into Neutrophil Extracellular Traps through Systematic Evaluation of Citrullination and Peptidylarginine Deiminases. J. Immunol. Res. 2019, 1–11. 10.1155/2019/2160192 PMC6434303 30993117

[B73] Hong C. Tontonoz P. (2008). Coordination of Inflammation and Metabolism by PPAR and LXR Nuclear Receptors. Curr. Opin. Genet. Dev. 18 (5), 461–467. 10.1016/j.gde.2008.07.016 18782619 PMC2641014

[B74] Huang H. (2018). Matrix Metalloproteinase-9 (MMP-9) as a Cancer Biomarker and MMP-9 Biosensors: Recent Advances. Sensors 18, 3249. 10.3390/s18103249 PMC6211011 30262739

[B75] Huh S. J. Liang S. Sharma A. Dong C. Robertson G. P. (2010). Transiently Entrapped Circulating Tumor Cells Interact with Neutrophils to Facilitate Lung Metastasis Development. Cancer Res. 70 (14), 6071–6082. 10.1158/0008-5472.can-09-4442 20610626 PMC2905495

[B76] Huo M. Wang L. Wang Y. Chen Y. Shi J. (2019). Nanocatalytic Tumor Therapy by Single-Atom Catalysts. ACS Nano 13 (2), 2643–2653. 10.1021/acsnano.9b00457 30753056

[B77] Hussain Y. Mirzaei S. Ashrafizadeh M. Zarrabi A. Hushmandi K. Khan H. (2021). Quercetin and Its Nano-Scale Delivery Systems in Prostate Cancer Therapy: Paving the Way for Cancer Elimination and Reversing Chemoresistance. Cancers (Basel) 13, 1602. 10.3390/cancers13071602 33807174 PMC8036441

[B78] Ingold I. Berndt C. Schmitt S. Doll S. Poschmann G. Buday K. (2018). Selenium Utilization by GPX4 Is Required to Prevent Hydroperoxide-Induced Ferroptosis. Cell 172 (3), 409–422.e21. 10.1016/j.cell.2017.11.048, 29290465

[B79] Kagan V. E. Mao G. Qu F. Angeli P. Doll S. Croix C. S. (2017). Oxidized Arachidonic and Adrenic PEs Navigate Cells to Ferroptosis. Nat. Chem. Biol. 13, 81–90. 10.1038/nchembio.2238 27842066 PMC5506843

[B80] Kaiser W. J. Upton J. W. Long A. B. Livingston-Rosanoff D. Daley-Bauer L. P. Hakem R. (2011). RIP3 Mediates the Embryonic Lethality of Caspase-8-Deficient Mice. Nature 471, 368–372. 10.1038/nature09857 21368762 PMC3060292

[B81] Karan D. Tawfik O. Dubey S. (2017). Expression Analysis of Inflammasome Sensors and Implication of NLRP12 Inflammasome in Prostate Cancer. Sci. Rep. 7 (1), 4378. 10.1038/s41598-017-04286-4 28663562 PMC5491527

[B82] Karki R. Sharma B. R. Lee E. Banoth B. Malireddi R. K. S. Samir P. (2020). Interferon Regulatory Factor 1 Regulates PANoptosis to Prevent Colorectal Cancer. JCI Insight 5 (12), e136720. 10.1172/jci.insight.136720 PMC7406299 32554929

[B83] Katarina D. Sanja K. Ivana M. K. Sotir S. Oliver S. Selim K. (2017). Comparative Proteomics Analysis of Urine Reveals Down-Regulation of Acute Phase Response Signaling and LXR/RXR Activation Pathways in Prostate Cancer. Proteomes 6 (1), 1. 10.3390/proteomes6010001 PMC5874760 29286311

[B84] Khan S. Jain M. Mathur V. Feroz S. M. (2016). Chronic Inflammation and Cancer: Paradigm on Tumor Progression, Metastasis and Therapeutic Intervention. Gulf J. Oncolog. 1 (20), 86–93. 27050184

[B85] Kharaziha P. Chioureas D. Baltatzis G. Fonseca P. Rodriguez P. Gogvadze V. (2015). Sorafenib-induced Defective Autophagy Promotes Cell Death by Necroptosis. Oncotarget 6 (35), 37066–37082. 10.18632/oncotarget.5797 26416459 PMC4741916

[B86] Kolaczkowska E. Jenne C. N. Surewaard B. G. J. Thanabalasuriar A. Lee W.-Y. Sanz M.-J. (2015). Molecular Mechanisms of NET Formation and Degradation Revealed by Intravital Imaging in the Liver Vasculature. Nat. Commun. 6, 6673. 10.1038/ncomms7673 25809117 PMC4389265

[B87] Kreuz S. Siegmund D. Scheurich P. Wajant H. (2001). NF-κB Inducers Upregulate cFLIP, a Cycloheximide-Sensitive Inhibitor of Death Receptor Signaling. Mol. Cel Biol. 21 (12), 3964–3973. 10.1128/mcb.21.12.3964-3973.2001 PMC87059 11359904

[B88] Lerman I. Hammes S. R. (2018). Neutrophil Elastase in the Tumor Microenvironment. Steroids 133, 96–101. 10.1016/j.steroids.2017.11.006 29155217 PMC5870895

[B89] Lerman I. Ma X. Seger C. Maolake A. Garcia-Hernandez M. L. Rangel-Moreno J. (2019). Epigenetic Suppression of SERPINB1 Promotes Inflammation-Mediated Prostate Cancer Progression. Mol. Cancer Res. 17 (4), 845–859. 10.1158/1541-7786.MCR-18-0638 30610107 PMC6445715

[B90] Li P. Wang D. Yao H. Doret P. Hao G. Shen Q. (2010). Coordination of PAD4 and HDAC2 in the Regulation of P53-Target Gene Expression. Oncogene 29 (21), 3153–3162. 10.1038/onc.2010.51 20190809 PMC2913128

[B91] Li X. Guo J. Ding A. P. Qi W. W. Zhang P. H. Lv J. (2017). Association of Mixed Lineage Kinase Domain-Like Protein Expression with Prognosis in Patients with Colon Cancer. Technol. Cancer Res. Treat. 16 (4), 428–434. 10.1177/1533034616655909 27432118 PMC5616063

[B92] Li J. Cao F. Yin H.-l. Huang Z.-j. Lin Z.-t. Mao N. (2020). Ferroptosis: Past, Present and Future. Cel Death Dis. 11 (2), 88. 10.1038/s41419-020-2298-2 PMC6997353 32015325

[B93] Lin H. Pan J. C. Zhang F. M. Huang B. Chen X. Zhuang J. T. (2015). Matrix Metalloproteinase-9 Is Required for Vasculogenic Mimicry by clear Cell Renal Carcinoma Cells. Urol. Oncol. 33 (4), 168.e9–168.e16. 10.1016/j.urolonc.2014.12.007 25618297

[B94] Lin L. S. Song J. Song L. Ke K. Liu Y. Zhou Z. (2018). Simultaneous Fenton-like Ion Delivery and Glutathione Depletion by MnO2 -Based Nanoagent to Enhance Chemodynamic Therapy. Angew. Chem. Int. Ed. Engl. 57 (18), 4902–4906. 10.1002/anie.201712027 29488312

[B95] Liu X. Lieberman J. (2017). How ICE Lights the Pyroptosis Fire. Cel Death Differ. 24 (2), 197–199. 10.1038/cdd.2016.157 PMC5299720 28060375

[B96] Liu X. Zhou M. Mei L. Ruan J. Hu Q. Peng J. (2016). Key Roles of Necroptotic Factors in Promoting Tumor Growth. Oncotarget 7 (16), 22219–22233. 10.18632/oncotarget.7924 26959742 PMC5008357

[B97] Liu M. R. Zhu W. T. Pei D. S. (2021). System Xc-: a Key Regulatory Target of Ferroptosis in Cancer. Invest. New Drugs 39 (5), 1123–1131. 10.1007/s10637-021-01070-0 33506324

[B98] Liu Y. (2006). Fatty Acid Oxidation Is a Dominant Bioenergetic Pathway in Prostate Cancer. Prostate Cancer Prostatic Dis. 9 (3), 230–234. 10.1038/sj.pcan.4500879 16683009

[B99] Longstaff C. Varjú I. Sótonyi P. Szabó L. Krumrey M. Hoell A. (2013). Mechanical Stability and Fibrinolytic Resistance of Clots Containing Fibrin, DNA, and Histones. J. Biol. Chem. 288 (10), 6946–6956. 10.1074/jbc.m112.404301 23293023 PMC3591605

[B100] Louandre C. Ezzoukhry Z. Godin C. Barbare J.-C. Mazière J. C. Chauffert B. (2013). Iron-dependent Cell Death of Hepatocellular Carcinoma Cells Exposed to Sorafenib. Int. J. Cancer 133 (7), 1732–1742. 10.1002/ijc.28159, 23505071

[B101] Lu Z. Wu C. Zhu M. Song W. Wang H. Wang J. (2020). Ophiopogonin D' Induces RIPK1-Dependent Necroptosis in Androgen Dependent LNCaP Prostate Cancer Cells. Int. J. Oncol. 56 (2), 439–447. 10.3892/ijo.2019.4945 31894265 PMC6959467

[B102] Mackenzie C. D. Geary T. G. (2011). Flubendazole: a Candidate Macrofilaricide for Lymphatic Filariasis and Onchocerciasis Field Programs. Expert Rev. anti-infect. Ther. 9 (5), 497–501. 10.1586/eri.11.30 21609260

[B103] Maiorino M. Conrad M. Ursini F. (2018). GPx4, Lipid Peroxidation, and Cell Death: Discoveries, Rediscoveries, and Open Issues. Antioxid. Redox Signal. 29 (1), 61–74. 10.1089/ars.2017.7115 28462584

[B104] Markowitsch S. D. Schupp P. Lauckner J. Vakhrusheva O. Slade K. S. Mager R. (2020). Artesunate Inhibits Growth of Sunitinib-Resistant Renal Cell Carcinoma Cells through Cell Cycle Arrest and Induction of Ferroptosis. Cancers 12 (11), 3150. 10.3390/cancers12113150 PMC7692972 33121039

[B105] Marmorstein R. (2004). Structure and Chemistry of the Sir2 Family of NAD+-Dependent Histone/protein Deactylases. Biochem. Soc. Trans. 32 (Pt 6), 904–909. 10.1042/BST0320904 15506920

[B106] Martinod K. Demers M. Fuchs T. A. Wong S. L. Brill A. Gallant M. (2013). Neutrophil Histone Modification by Peptidylarginine Deiminase 4 Is Critical for Deep Vein Thrombosis in Mice. Proc. Natl. Acad. Sci. 110 (21), 8674–8679. 10.1073/pnas.1301059110 23650392 PMC3666755

[B107] Mcdonald B. Spicer J. Giannais B. Fallavollita L. Brodt P. Ferri L. E. (2010). Systemic Inflammation Increases Cancer Cell Adhesion to Hepatic Sinusoids by Neutrophil Mediated Mechanisms. Int. J. Cancer 125 (6), 1298–1305. 10.1002/ijc.24409 19431213

[B108] Mego M. Ranieri E. Feng L. Zhang K. Ma J. (2019). Cooperation between the Inflammation and Coagulation Systems Promotes the Survival of Circulating Tumor Cells in Renal Cell Carcinoma Patients. Front. Oncol. 9 (2019), 504. 10.3389/fonc.2019.00504 31263677 PMC6590108

[B109] Michan S. Sinclair D. (2007). Sirtuins in Mammals: Insights into Their Biological Function. Biochem. J. 404 (1), 1–13. 10.1042/bj20070140 17447894 PMC2753453

[B110] Miess H. Dankworth B. Gouw A. M. Rosenfeldt M. Schmitz W. Jiang M. (2018). The Glutathione Redox System Is Essential to Prevent Ferroptosis Caused by Impaired Lipid Metabolism in clear Cell Renal Cell Carcinoma. Oncogene 37, 5435–5450. 10.1038/s41388-018-0315-z 29872221 PMC6173300

[B111] Mirzaei S. Gholami M. H. Mahabady M. K. Nabavi N. Zabolian A. Banihashemi S. M. (2021a). Pre-clinical Investigation of STAT3 Pathway in Bladder Cancer: Paving the Way for Clinical Translation. Biomed. Pharmacother. 133 (2021), 111077. 10.1016/j.biopha.2020.111077 33378975

[B112] Mirzaei S. Paskeh M. D. A. Hashemi F. Zabolian A. Hashemi M. Entezari M. (2021b). Long Non-coding RNAs as New Players in Bladder Cancer: Lessons from Pre-clinical and Clinical Studies. Life Sci., 119948. 10.1016/j.lfs.2021.119948 34520771

[B113] Mizuno R. Kawada K. Itatani Y. Ogawa R. Kiyasu Y. Sakai Y. (2019). The Role of Tumor-Associated Neutrophils in Colorectal Cancer. Int. J. Mol. Sci. 20 (3), 529. 10.3390/ijms20030529 PMC6386937 30691207

[B114] Moriai S. Takahara M. Ogino T. Nagato T. Kishibe K. Ishii H. (2009). Production of Interferon-γ-Inducible Protein-10 and Its Role as an Autocrine Invasion Factor in Nasal Natural Killer/T-Cell Lymphoma Cells. Clin. Cancer Res. 15 (22), 6771–6779. 10.1158/1078-0432.ccr-09-1052 19887486

[B115] Mou Y. Wu J. Zhang Y. Abdihamid O. Li B. (2021). Low Expression of Ferritinophagy-Related NCOA4 Gene in Relation to Unfavorable Outcome and Defective Immune Cells Infiltration in clear Cell Renal Carcinoma. BMC Cancer 21 (1), 18. 10.1186/s12885-020-07726-z 33402128 PMC7786469

[B116] Murphy J. M. Czabotar P. E. Hildebrand J. M. Lucet I. S. Zhang J. G. Alvarez-Diaz S. (2018). The Pseudokinase MLKL Mediates Necroptosis via a Molecular Switch Mechanism. Immunity 39 (3), 443–453. 10.1016/j.immuni.2013.06.018 24012422

[B117] Najmeh S. Cools-Lartigue J. Rayes R. F. Gowing S. Vourtzoumis P. Bourdeau F. (2017). Neutrophil Extracellular Traps Sequester Circulating Tumor Cells via 1-integrin Mediated Interactions. Int. J. Cancer 140 (10), 2321–2330. 10.1002/ijc.30635 28177522

[B118] Nakazawa D. Tomaru U. Yamamoto C. Jodo S. Ishizu A. (2012). Abundant Neutrophil Extracellular Traps in Thrombus of Patient with Microscopic Polyangiitis. Front. Immun. 3 (4), 333. 10.3389/fimmu.2012.00333 PMC3495275 23162551

[B119] Nassar Z. D. Mah C. Y. Dehairs J. Burvenich I. J. Butler L. M. (2020). Human DECR1 Is an Androgen-Repressed Survival Factor that Regulates PUFA Oxidation to Protect Prostate Tumor Cells from Ferroptosis. Elife. 9, e54166. 10.7554/eLife.54166 32686647 PMC7386908

[B120] Nugues A.-L. El Bouazzati H. Hétuin D. Berthon C. Loyens A. Bertrand E. (2014). RIP3 Is Downregulated in Human Myeloid Leukemia Cells and Modulates Apoptosis and Caspase-Mediated p65/RelA Cleavage. Cel Death Dis. 5 (8), e1384. 10.1038/cddis.2014.347 PMC4454320 25144719

[B121] Ording A. G. Horváth-Puhó E. Lash T. L. Ehrenstein V. Borre M. Vyberg M. (2015). Prostate Cancer, Comorbidity, and the Risk of Venous Thromboembolism: A Cohort Study of 44,035 Danish Prostate Cancer Patients, 1995-2011. Cancer 121 (20), 3692–3699. 10.1002/cncr.29535 26149752

[B122] Orning P. Weng D. Starheim K. Ratner D. Best Z. Lee B. (2018). Pathogen Blockade of TAK1 Triggers Caspase-8-dependent Cleavage of Gasdermin D and Cell Death. Science 362, 1064–1069. 10.1126/science.aau2818 30361383 PMC6522129

[B123] Ou W. Mulik R. S. Anwar A. Mcdonald J. G. He X. Corbin I. R. (2017). Low-density Lipoprotein Docosahexaenoic Acid Nanoparticles Induce Ferroptotic Cell Death in Hepatocellular Carcinoma. Free Radic. Biol. Med. 112 (1), 597–607. 10.1016/j.freeradbiomed.2017.09.002 28893626 PMC5848495

[B124] Ou Y. C. Li J. R. Wang J. D. Chen W. Y. Kuan Y. H. Yang C. P. (2018). Aspirin Restores ABT-737-Mediated Apoptosis in Human Renal Carcinoma Cells. Biochem. Biophys. Res. Commun. 502 (2), 187–193. 10.1016/j.bbrc.2018.05.142 29792865

[B125] Park J. Wysocki R. W. Amoozgar Z. Maiorino L. Fein M. R. Jorns J. (2016). Cancer Cells Induce Metastasis-Supporting Neutrophil Extracellular DNA Traps. Sci. Transl Med. 8 (361), 361ra138. 10.1126/scitranslmed.aag1711 PMC5550900 27798263

[B126] Peng J. Jiang H. Guo J. Huang J. Yuan Q. Xie J. (2020). CD147 Expression Is Associated with Tumor Proliferation in Bladder Cancer via GSDMD. Biomed. Res. Int. 2020 (5), 1–7. 10.1155/2020/7638975 PMC7054768 32149134

[B127] Pieterse E. Rother N. Garsen M. Hofstra J. M. Satchell S. C. Hoffmann M. (2017). Neutrophil Extracellular Traps Drive Endothelial-To-Mesenchymal Transition. Atvb 37 (7), 1371–1379. 10.1161/atvbaha.117.309002 28495931

[B128] Place D. E. Lee S. Kanneganti T.-D. (2021). PANoptosis in Microbial Infection. Curr. Opin. Microbiol. 59, 42–49. 10.1016/j.mib.2020.07.012 32829024 PMC7438227

[B129] Poli G. Fabi C. Bellet M. M. Costantini C. Nunziangeli L. Romani L. (2020). Epigenetic Mechanisms of Inflammasome Regulation. Ijms 21 (16), 5758. 10.3390/ijms21165758 PMC7460952 32796686

[B130] Qin Z. Ou S. Xu L. Sorensen K. Zhang Y. Hu D-P. (2021). Design and Synthesis of Isothiocyanate‐containing Hybrid Androgen Receptor (AR) Antagonist to Downregulate AR and Induce Ferroptosis in GSH–Deficient Prostate Cancer Cells. Chem. Biol. Drug Des. 97 (5), 1059–1078. 10.1111/cbdd.13826 33470049 PMC8168342

[B131] Quarato G. Guy C. S. Grace C. R. Llambi F. Nourse A. Rodriguez D. A. (2016). Sequential Engagement of Distinct MLKL Phosphatidylinositol-Binding Sites Executes Necroptosis. Mol. Cel. 61, 589–601. 10.1016/j.molcel.2016.01.011 PMC4769881 26853145

[B132] Rathinam V. A. K. Fitzgerald K. A. (2016). Inflammasome Complexes: Emerging Mechanisms and Effector Functions. Cell 165 (4), 792–800. 10.1016/j.cell.2016.03.046 27153493 PMC5503689

[B133] Reggiani F. Labanca V. Mancuso P. Rabascio C. Talarico G. Orecchioni S. (2017). Adipose Progenitor Cell Secretion of GM-CSF and MMP9 Promotes a Stromal and Immunological Microenvironment that Supports Breast Cancer Progression. Cancer Res. 77 (18), 5169–5182. 10.1158/0008-5472.CAN-17-0914 28754674

[B134] Reichert C. O. de Freitas F. A. Sampaio-Silva J. Rokita-Rosa L. Barros P. L. Levy D. (2020). Ferroptosis Mechanisms Involved in Neurodegenerative Diseases. Int. J. Mol. Sci. 21 (22), 1–27. 10.3390/ijms21228765 PMC7699575 33233496

[B135] Rouault T. A. (2005). The Intestinal Heme Transporter Revealed. Cell 122 (5), 649–651. 10.1016/j.cell.2005.08.027 16143096

[B136] Sánchez-Gastaldo A. Kempf E. González del Alba A. Duran I. (2017). Systemic Treatment of Renal Cell Cancer: A Comprehensive Review. Cancer Treat. Rev. 60, 77–89. 10.1016/j.ctrv.2017.08.010 28898679

[B137] Saluja M. Gilling P. (2017). Intravesical Bacillus Calmette-Guérin Instillation in Non-muscle-invasive Bladder Cancer: A Review. Int. J. Urol. 25 (1), 18–24. 10.1111/iju.13410 28741703

[B138] Sarhan J. Liu B. C. Muendlein H. I. Li P. Nilson R. Tang A. Y. (2018). Caspase-8 Induces Cleavage of Gasdermin D to Elicit Pyroptosis during Yersinia Infection. Proc. Natl. Acad. Sci. U S A. 115 (46), E10888. 10.1073/pnas.1809548115 30381458 PMC6243247

[B139] Sawada N. Inoue M. Iwasaki M. Sasazuki S. Shimazu T. Yamaji T. (2012). Consumption of N-3 Fatty Acids and Fish Reduces Risk of Hepatocellular Carcinoma. Gastroenterology 142 (7), 1468–1475. 10.1053/j.gastro.2012.02.018 22342990

[B140] Seiler A. Schneider M. Förster H. Roth S. Wirth E. K. Culmsee C. (2008). Glutathione Peroxidase 4 Senses and Translates Oxidative Stress into 12/15-Lipoxygenase Dependent- and AIF-Mediated Cell Death. Cel Metab. 8 (3), 237–248. 10.1016/j.cmet.2008.07.005 18762024

[B141] Shinde-Jadhav S. Mansure J. J. Rayes R. F. Marcq G. Ayoub M. Skowronski. R. (2021). Role of Neutrophil Extracellular Traps in Radiation Resistance of Invasive Bladder Cancer. Nat. Commun. 12 (1), 2776. 10.1038/s41467-021-23086-z 33986291 PMC8119713

[B142] Siegel R. L. Miller K. D. Jemal A. (2017). Cancer Statistics, 2017. CA: a Cancer J. Clin. 67 (1), 7–30. 10.3322/caac.21387 28055103

[B143] Siegel R. L. Miller K. D. Jemal A. (2020). Cancer Statistics, 2020. CA A. Cancer J. Clin. 70 (1), 7–30. 10.3322/caac.21590 31912902

[B144] Sonkusre P. Cameotra S. S. (2017). Biogenic Selenium Nanoparticles Induce ROS-Mediated Necroptosis in PC-3 Cancer Cells through TNF Activation. J. Nanobiotechnol. 15 (1), 43. 10.1186/s12951-017-0276-3 PMC5463494 28592284

[B145] Straif K. Benbrahim-Tallaa L. Baan R. Grosse Y. Secretan B. El Ghissassi F. (2009). A Review of Human Carcinogens--Part C: Metals, Arsenic, Dusts, and Fibres. Lancet Oncol. 10, 453–454. 10.1016/s1470-2045(09)70134-2 19418618

[B174] Sun Z. Yang P. (2004). Role of Imbalance between Neutrophil Elastase and Alpha 1-Antitrypsin in Cancer Development and Progression. Lancet Oncol. 5 (3), 182–190. 10.1016/S1470-2045(04)01414-7 15003202

[B146] Swanson K. V. Deng M. Ting P. Y. (2019). The NLRP3 Inflammasome: Molecular Activation and Regulation to Therapeutics. Nat. Rev. Immunol. 19, 477–489. 10.1038/s41577-019-0165-0 31036962 PMC7807242

[B147] Takeuchi O. Akira S. (2010). Pattern Recognition Receptors and Inflammation. Cell 140 (6), 805–820. 10.1016/j.cell.2010.01.022 20303872

[B148] Tan Y. F. Wang M. Chen Z. Y. Wang L. Liu X. H. (2020). Inhibition of BRD4 Prevents Proliferation and Epithelial-Mesenchymal Transition in Renal Cell Carcinoma via NLRP3 Inflammasome-Induced Pyroptosis. Cel Death Dis. 11 (4), 239. 10.1038/s41419-020-2431-2 PMC7165180 32303673

[B149] Tang D. Kroemer G. (2020). Ferroptosis. Curr. Biol. 30 (21), R1292–R1297. 10.1016/j.cub.2020.09.068 33142092

[B150] Tang J. Bei M. Zhu J. Xu G. Chen D. Jin X. (2021). Acute Cadmium Exposure Induces GSDME-Mediated Pyroptosis in Triple-Negative Breast Cancer Cells through ROS Generation and NLRP3 Inflammasome Pathway Activation. Environ. Toxicol. Pharmacol. 87, 103686. 10.1016/j.etap.2021.103686 34098069

[B151] Thomas G. M. Brill A. Crescence L. Gallant M. Dubois C. Wagner D. D. (2015). Tissue Factor Expressed by Circulating Cancer Cell-Derived Microparticles Drastically Increases the Incidence of Deep Vein Thrombosis in Mice. J. Thromb. Haemost. 13 (7), 1310–1319. 10.1111/jth.13002 25955268 PMC4496280

[B152] Tian X. Zhang S. Zhang Q. Kang L. Shen Y. (2020). Resveratrol Inhibits Tumor Progression by Down-Regulation of NLRP3 in Renal Cell Carcinoma. J. Nutr. Biochem. 85, 108489. 10.1016/j.jnutbio.2020.108489 32827663

[B153] Ting A. T. Bertrand M. J. M. (2016). More to Life Than NF-κB in TNFR1 Signaling. Trends Immunol. 37, 535–545. 10.1016/j.it.2016.06.002 27424290 PMC5076853

[B154] Tohme S. Yazdani H. O. Al-Khafaji A. B. Chidi A. P. Loughran P. Mowen K. (2016). Neutrophil Extracellular Traps Promote the Development and Progression of Liver Metastases after Surgical Stress. Cancer Res. 76 (6), 1367–1380. 10.1158/0008-5472.CAN-15-1591 26759232 PMC4794393

[B155] Viganò E. Mortellaro A. (2013). Caspase-11: the Driving Factor for Noncanonical Inflammasomes. Eur. J. Immunol. 43 (9), 2240–2245. 10.1002/eji.201343800 24037676

[B156] Wang J. Lu Z. Kong Y. Wei S. He W. Xu H. (2017a). Ophiopogonin D' Induces Necroptosis in Prostate Cancer PC3 Cells through RIP1/MLKL Pathway. J. Third Mil. Med. Univ. 39 (3), 201–207. 10.16016/j.1000-5404.201609037

[B157] Wang Y. Gao W. Shi X. Ding J. Liu W. He H. (2017b). Chemotherapy Drugs Induce Pyroptosis through Caspase-3 Cleavage of a Gasdermin. Nature 547 (7661), 99–103. 10.1038/nature22393 28459430

[B158] Wang Y. Yin B. Li D. Wang G. Han X. Sun X. (2018). GSDME Mediates Caspase-3-dependent Pyroptosis in Gastric Cancer. Biochem. Biophys. Res. Commun. 495 (1), 1418–1425. 10.1016/j.bbrc.2017.11.156 29183726

[B159] Wang K. Xu T. Ruan H. Xiao H. Liu J. Song Z. (2019a). LXRα Promotes Cell Metastasis by Regulating the NLRP3 Inflammasome in Renal Cell Carcinoma. Cel Death Dis. 10 (3), 159. 10.1038/s41419-019-1345-3 PMC6377709 30770793

[B160] Wang W. Green M. Choi J. E. Gijón M. Kennedy P. D. Johnson J. K. (2019b). CD8+ T Cells Regulate Tumour Ferroptosis during Cancer Immunotherapy. Nature 569 (7755), 270–274. 10.1038/s41586-019-1170-y 31043744 PMC6533917

[B161] Wang K.-j. Meng X.-y. Chen J.-f. Wang K.-y. Zhou C. Yu R. (2021). Emodin Induced Necroptosis and Inhibited Glycolysis in the Renal Cancer Cells by Enhancing ROS. Oxid. Med. Cell. Longev. 2021, 1–17. 10.1155/2021/8840590 PMC7837784 33532038

[B162] Wei C. Hong-Da C. Yi-Wen Y. Ni L. Wan-Qing C. (2021). Changing Profiles of Cancer burden Worldwide and in China: a Secondary Analysis of the Global Cancer Statistics 2020. Chin. Med. J. 134 (07), 783–791. 10.1097/cm9.0000000000001474 33734139 PMC8104205

[B163] Weigand I. Schreiner J. Röhrig F. Sun N. Landwehr L. S. Urlaub H. (2020). Active Steroid Hormone Synthesis Renders Adrenocortical Cells Highly Susceptible to Type II Ferroptosis Induction. Cel Death Dis. 11 (3), 192. 10.1038/s41419-020-2385-4 PMC7078189 32184394

[B164] Wilhelm S. M. Carter C. Tang L. Wilkie D. McNabola A. Rong H. (2004). BAY 43-9006 Exhibits Broad Spectrum Oral Antitumor Activity and Targets the RAF/MEK/ERK Pathway and Receptor Tyrosine Kinases Involved in Tumor Progression and Angiogenesis. Cancer Res. 64 (19), 7099–7109. 10.1158/0008-5472.can-04-1443 15466206

[B165] Wilson K. P. Black J-A. Thomson J. A. Kim E. E. Griffith J. P. Navia M. A. (1994). Structure and Mechanism of Interleukin-1beta Converting Enzyme. Nature 370 (6487), 270–275. 10.1038/370270a0 8035875

[B166] Wilson T. J. Nannuru K. C. Futakuchi M. Singh R. K. (2010). Cathepsin G-Mediated Enhanced TGF-β Signaling Promotes Angiogenesis via Upregulation of VEGF and MCP-1. Cancer Lett. 288 (2), 162–169. 10.1016/j.canlet.2009.06.035 19646811 PMC2815079

[B167] Wong W. W.-L. Gentle I. E. Nachbur U. Anderton H. Vaux D. L. Silke J. (2010). RIPK1 Is Not Essential for TNFR1-Induced Activation of NF-κB. Cel Death Differ. 17 (3), 482–487. 10.1038/cdd.2009.178 19927158

[B168] Woo J. H. Shimoni Y. Yang W. S. Subramaniam P. Iyer A. Nicoletti P. (2015). Elucidating Compound Mechanism of Action by Network Perturbation Analysis. Cell 162 (2), 441–451. 10.1016/j.cell.2015.05.056 26186195 PMC4506491

[B169] Wu G. J. Bao J. S. Yue Z. J. Zeng F. C. Cen S. Tang Z. Y. (2018). Elevated Expression of Matrix Metalloproteinase-9 Is Associated with Bladder Cancer Pathogenesis. J. Cancer Res. Ther. 14, S54. 10.4103/0973-1482.163761 29578150

[B170] Wu L. Saxena S. Awaji M. Singh R. K. (2019). Tumor-Associated Neutrophils in Cancer: Going Pro. Cancers 11 (4), 564. 10.3390/cancers11040564 PMC6520693 31010242

[B171] Wu G. Wang Q. Xu Y. Li Q. Cheng L. (2020). A New Survival Model Based on Ferroptosis-Related Genes for Prognostic Prediction in clear Cell Renal Cell Carcinoma. Aging (Albany NY) 12 (14), 14933–14948. 10.18632/aging.103553 32688345 PMC7425493

[B172] Yagoda N. von Rechenberg M. Zaganjor E. Bauer A. J. Yang W. S. Fridman D. J. (2007). RAS-RAF-MEK-dependent Oxidative Cell Death Involving Voltage-dependent Anion Channels. Nature 447 (7146), 865–869. 10.1038/nature05859 PMC3047570 17568748

[B173] Yang W. S. Stockwell B. R. (2008). Synthetic Lethal Screening Identifies Compounds Activating Iron-dependent, Nonapoptotic Cell Death in Oncogenic-RAS-Harboring Cancer Cells. Chem. Biol. 15 (3), 234–245. 10.1016/j.chembiol.2008.02.010 18355723 PMC2683762

[B175] Yang W. S. Sriramaratnam R. Welsch M. E. Shimada K. Skouta R. Viswanathan V. S. (2014). Regulation of Ferroptotic Cancer Cell Death by GPX4. Cell 156 (1-2), 317–331. 10.1016/j.cell.2013.12.010 24439385 PMC4076414

[B176] Yang W. S. Kim K. J. Gaschler M. M. Patel M. Shchepinov M. S. Stockwell B. R. (2016). Peroxidation of Polyunsaturated Fatty Acids by Lipoxygenases Drives Ferroptosis. Proc. Natl. Acad. Sci. U S A. 113 (34), E4966–E4975. 10.1073/pnas.1603244113 27506793 PMC5003261

[B177] Yang J. Liu Z. Wang C. Yang R. Rathkey J. K. Pinkard O. W. (2018). Mechanism of Gasdermin D Recognition by Inflammatory Caspases and Their Inhibition by a Gasdermin D-Derived Peptide Inhibitor. Proc. Natl. Acad. Sci. U S A. 115 (26), 6792–6797. 10.1073/pnas.1800562115 29891674 PMC6042100

[B178] Yang Y. Liu T. Hu C. Xia H. Zhao L. (2021). Ferroptosis Inducer Erastin Downregulates Androgen Receptor and its Splice Variants in Castrationresistant Prostate Cancer. Oncol. Rep. 45 (4), 25. 10.3892/or.2021.7976 33649848

[B179] Yant L. J. Ran Q. Rao L. Van Remmen H. Shibatani T. Belter J. G. (2003). The Selenoprotein GPX4 Is Essential for Mouse Development and Protects from Radiation and Oxidative Damage Insults. Free Radic. Biol. Med. 34 (4), 496–502. 10.1016/s0891-5849(02)01360-6 12566075

[B180] Yeung C. Dinh T. Lee J. (2014). The Health Economics of Bladder Cancer: an Updated Review of the Published Literature. PharmacoEconomics 32 (11), 1093–1104. 10.1007/s40273-014-0194-2 25056838

[B181] Zhai E. Chen J. Kang W. Ye Z. Cai S. (2016). Prognostic Value of Mixed Lineage Kinase Domain-like Protein Expression in the Survival of Patients with Gastric Caner. Tumor Biol. 37 (10), 13679–13685. 10.1007/s13277-016-5229-1 27473085

[B182] Zhao C. Zhou Y. Ran Q. Yao Y. Zhang H. Ju J. (2020). MicroRNA-381-3p Functions as a Dual Suppressor of Apoptosis and Necroptosis and Promotes Proliferation of Renal Cancer Cells. Front Cel Dev Biol. 8, 290. 10.3389/fcell.2020.00290 PMC7198711 32411707

[B183] Zhou Y. An L. L. Chaerkady R. Mittereder N. Clarke L. Cohen T. S. (2018). Evidence for a Direct Link between PAD4-Mediated Citrullination and the Oxidative Burst in Human Neutrophils. Sci. Rep. 8 (1), 15228. 10.1038/s41598-018-33385-z 30323221 PMC6189209

[B184] Zhou X. Zou L. Chen W. Yang T. Luo J. Wu K. (2020). Flubendazole, FDA-Approved Anthelmintic, Elicits Valid Antitumor Effects by Targeting P53 and Promoting Ferroptosis in Castration-Resistant Prostate Cancer. Pharmacol. Res. 164, 105305. 10.1016/j.phrs.2020.105305 33197601

[B185] Zou Y. Palte M. J. Deik A. A. Li H. Eaton J. K. Wang W. (2019). A GPX4-dependent Cancer Cell State Underlies the clear-cell Morphology and Confers Sensitivity to Ferroptosis. Nat. Commun. 10 (1), 1617. 10.1038/s41467-019-09277-9 30962421 PMC6453886

[B186] Zychlinsky A. Prevost M. C. Sansonetti P. J. (1992). Shigella Flexneri Induces Apoptosis in Infected Macrophages. Nature 358 (6382), 167–169. 10.1038/358167a0 1614548

